# Somatosensory control of thalamic relay neurons is regulated by two distinct layer 6 feedback systems

**DOI:** 10.1016/j.isci.2025.114427

**Published:** 2025-12-12

**Authors:** Josephine Ansorge, Denise Manahan-Vaughan

**Affiliations:** 1 Ruhr University Bochum, Faculty of Medicine, Department of Neurophysiology, Bochum, Germany

**Keywords:** Biological sciences, Natural sciences, Neuroscience, Sensory neuroscience, Systems neuroscience

## Abstract

The medial posterior (POm) and ventral posterior medial (VPM) thalamic nuclei sort and filter somatosensory information that is sent to the primary somatosensory cortex (S1). Corticothalamic feedback from distinct cell populations of S1 layer 6 (S1L6) targets both POm and VPM. To differentiate their roles, electrophysiological recordings were conducted in awake mice during the photoinactivation of S1L6B-Drd1-expressing neurons (that project to POm), or S1L6-Ntsr1-expressing neurons (that project to POm and VPM). Photoinactivation of either pathway suppressed POm activity when whiskers were deflected, regardless of whisking state, but S1L6-Ntsr1-POm inputs were most affected when whisker deflection occurred during non-whisking. Conversely, S1L6-Ntsr1-VPM inputs responded to whisker deflections during whisking. We identified a novel projection from S1L6-Drd1-expressing neurons to the zona incerta, indicating a role for this GABAergic structure in a corticothalamic circuit that modulates POm. These results indicate corticothalamic control of somatosensory thalamus is regulated, in a context-dependent manner, by two distinct S1L6 feedback systems supporting precision and fine-tuning of somatosensory perception.

## Highlights

• S1L6 Drd1 and Ntsr1-expressing cells were inhibited optogenetically during whisking in mice
• Specific roles for S1L6-thalamic inputs to POm/VPM during whisker deflection were identified
• Neuroanatomical tracing revealed a projection from S1L6 Drd1-neurons to zona incerta
• Drd1/Ntsr1 neurons regulate somatosensory thalamus in a nucleus and context-dependent manner

## Graphical abstract

**Figure:**
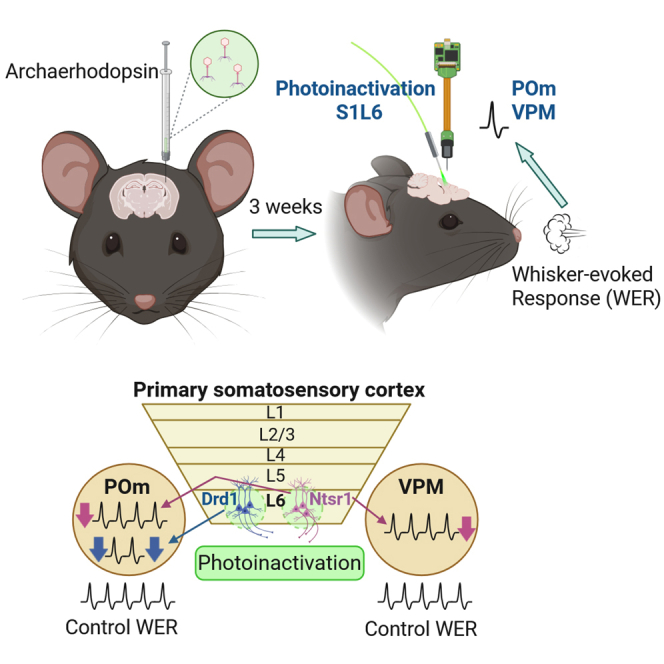

## Introduction

In rodents and whiskered (vibrissae-possessing) mammals, tactile information is transmitted from the whiskers, via the brainstem and thalamus, to the somatosensory cortex (S1). The somatosensory system is characterized by the conserved hierarchy of projections from the whiskers, that are processed by different populations of thalamic relay neurons, including the ventral posterior medial thalamus (VPM), the ventral posterior lateral nucleus (VPL) and the posterior medial thalamus (POm).^1^ Whereas VPM receives somatosensory information from the face and neck, the VPL receives information from the rest of the body.^1^ VPM and VPL are regarded as first order nuclei that convey somatosensory stimuli to the cortex. With regard to the vibrissae, neurons of the lemniscal pathway receive input from one single whisker and are organized into “barrelettes” at the level of trigeminal nuclei in the brain stem.^2^ These neurons project, in turn, to the dorsal medial region of the VPM, which is organized into “barreloids.”^3^ Afferents from the VPM project to S1, that is organized into anatomical units called “barrels,”^4^ and through this conserved projection system, information from a single whisker can be transmitted to its corresponding barrel in S1.^5^

Within S1, information is extracted about item dimensions and localization, along with dynamic representations of ongoing alterations in tactile space and/or tactile input^6^^,^^7^ that subsequently, and with the support of structures such as the retrosplenial cortex,^8^ are integrated into detailed and dynamic sensory representations of features and dimensions of space. The thalamus subserves a key role in the transmission of tactile information to S1^9^ and does so by transmitting tactile information received from the whiskers via the abovementioned lemniscal pathway, as well as via two additional pathways to S1. First-order information about sensory perception is enabled by afferent projections from the VPM, predominantly to layers 4–6 of S1.^5^^,^^10^ This provides information, for example, about object characterization that can even be generated based on the stimulation of just one whisker.^11^ *Temporal* information about object localization is transmitted by the extralemniscal pathway that is also mediated by the VPM.^3^ Finally, paralemniscal projections, which provide information about whisker kinematics,^12^ including the deflection patterns of multiples of whiskers,^2^ are delivered to POm.^13^

POm has been described as a higher-order relay structure of the thalamus^14^ that integrates corticofugal, motor, and tactile information^15^ and serves to optimize tactile signal-to-noise ratios by helping to discriminate subthreshold from suprathreshold stimuli.^16^ These properties, in turn, may enable context-dependent information processing by POm,^17^ related to the processing of stimulus-reward association, anticipated reward delay, and reward saliency.^18^ In line with these possibilities, activity in POm can prolong cortical responses in S1 layer (L)5, a process that may subserve the prioritization of salient tactile information by S1. The question arises as to whether this is a passive process, meaning that increases in signal intensity and frequency arriving in POm result in an unadulterated increase in signal intensity and frequency arriving in S1, or whether POm undergoes *dynamic* modulation that promotes experience- and context-dependent interpretations, by S1, of these properties. The latter possibility is supported by evidence that thalamic nuclei can engage in experience-dependent modifications that assist the creation of dynamic functional representations of stimulus salience.^19^^,^^20^ Furthermore, corticofugal projections from S1L5 target single cells within POm,^21^ suggesting that finely tuned the modulation of POm neuronal responses is likely. Recently, it was proposed that S1L6 may support the generation of stimulus predictions^22^ that, in turn, could facilitate the context-dependent processing of tactile information.^17^

Although the role of cortical microcircuitry in predictive coding has been studied and characterized in detail,^23^^,^^24^^,^^25^ knowledge about thalamocortical circuitry and the role of vibrissal corticothalamocortical circuit loops in these processes remains limited. VPM sends afferents primarily to S1L4, as well as to S1L2/3 and S1L5, and S1L6.^26^ POm sends afferents to S1L1, L2, and L5.^5^^,^^16^ It instigates direct excitation of S1L2 and S1L5,^27^^,^^28^ including synaptic plasticity in S1L2^29^ that may support intracortical plasticity by means of disinhibition.^30^ By contrast, it has been proposed that VPM does not support experience-dependent cortical plasticity.^31^

Corticothalamic pathways have been identified that extend from S1L6 to the thalamus,^32^^,^^33^ one of which extends *specifically* from layer 6B of S1 to POm.^32^^,^^34^ S1 is composed of roughly 85% excitatory spiny stellate, star pyramidal, and pyramidal cells and ca. 15% inhibitory interneurons.^35^ Corticothalamic projections originate from pyramidal cells in L6A, whereas corticothalamic projections from L6B derive from both spiny stellate cells and pyramidal cells.^33^^,^^35^ An excitatory S1L6B projection to POm emanates from cells that express the dopamine D1 receptor (Drd1).^34^ Another excitatory cell population in S1L6, that expresses the neurotensin receptor 1 (Ntsr1), projects to VPM, POm, and the reticular thalamic nucleus (TRN).^36^ Both Drd1 and Ntrsr1-expressing cell populations contribute to the corticothalamic regulation of somatosensory thalamic relay cell output^32^^,^^37^ and it has been reported that Ntsr1-expressing S1L6 cells support early postnatal tuning of somatosensory responses.^33^ To what extent these differentiated corticothalamic projections from S1L6 dynamically influence electrophysiological firing characteristics in adult POm and VPM, thereby putatively supporting the fine-tuning of whisking perception and the acquisition of salient somatosensory information about object identity, is, as yet, unknown.

To address this question, we optogenetically targeted Drd1and Ntrsr1-expressing neuronal populations in S1L6 by transfecting an adeno-associated virus (AAV) carrying archaerhodopsin into two transgenic mouse lines that express a Cre-driver for Drd1 or Ntsr1. We recorded electrophysiologically from POm, VPM, and S1 during the photo-inhibition of Drd1, or Ntrsr1-expressing neurons in S1L6 and used this approach to compare the activity of POm and VPM in somatosensory perception during whisker deflection in whisking and non-whisking states. We report here that corticothalamic projections from Drd1 and Ntrsr1-expressing cell populations in S1L6 exert a functionally distinct feedback modulation of context-dependent somatosensory information processing in POm and VPM. We propose that these discrete corticothalamic pathways from S1L6 to these thalamic areas enable differentiated forms of experience- and context-dependent modulation of information transfer from the thalamus to the somatosensory cortex.

## Results

### Cortical and thalamic spiking activity in awake head-restrained mice differs depending on whisking state

**Figure 1:**
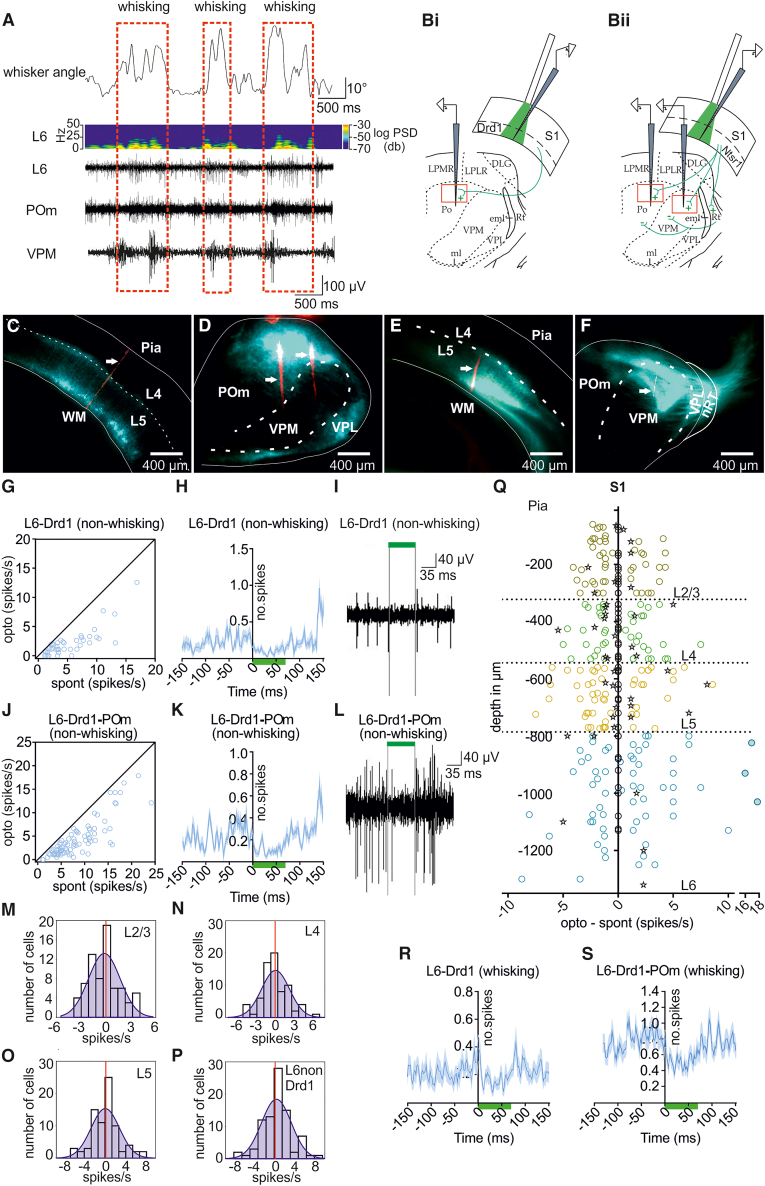
Cortical and thalamic spiking activity in awake head-restrained mice differs depending on whisking state (A) Top panel: Whisker tracking in awake head-restrained mice in combination with whisking (red rectangles) and non-whisking episodes. Middle panel: sliding window fast Fourier transformation of local field potentials recorded in S1L6 during whisker tracking. Lower panels: *In vivo* electrophysiology recordings of an S1L6 unit, a POm unit, and a VPM unit, recorded simultaneously during whisker tracking. (Bi-Bii) Scheme of the recording positions in the barrel cortex and thalamus for the Drd1-cre (Bi) and Ntsr1-cre (Bii) mice. The red rectangle in POm and VPM shows the relative location of the recorded thalamic areas (scheme adapted from the Franklin and Paxinos mouse brain atlas.^38^ (C–F) Virus expression in Drd1-cre and Ntsr1-cre mice. S1L6-Drd1 cells (C) express GFP-labelled archaerhodopsin. The arrow shows the electrode tract. (D) Same animal as in C, showing the axonal fluorescence from labeled S1L6- Drd1-expressing cells projecting to POm. (E) L6-Ntsr1 cells express GFP-labelled archaerhodopsin. The arrow shows the electrode tract. (F) Same animal as in E shows the axonal fluorescence from labeled L6-Ntsr1 cells projecting to VPM, POm, and nRT. (G–S) Inactivation of L6-Drd1 cells with light pulses (70 ms, 35.83 mW/mm^2^) and the effect on spontaneous activity during non-whisking (G–Q) and whisking (R–S). (G) Cells classified as S1L6- Drd1 cells (*n* = 46). Spontaneous activity, in the absence of light pulses (“spont”), is plotted against spiking activity during the light pulse (“opto”). (H) The averaged PSTHs (average in dark blue with SEM confidence bands) of all 46 recorded S1L6- Drd1 cells during photoinactivation (green bar) starting at time zero. (I) A representative *in vivo* electrophysiological recording of an S1L6- Drd1 cell during light pulse application. (J) Same plot as (G) for POm. (K) The averaged PSTHs of all 98 POm cells during the light pulse. (L) A representative *in vivo* electrophysiological recording of a POm cell, during the light pulse. (M–Q) The effect of S1L6- Drd1 inactivation on spontaneous activity in the other cortical layers. (M–P) Histograms of all recorded cells/cortical layers. X axis: “opto” minus “spont,” calculated in a 70 ms time window. (Q) Each cell from M-P is plotted separately at the recorded depth (y axis); x axis: “Opto” minus “Spont”; interneurons marked with stars; filled dots cells were classified as outliers (ROUT, Q = 1%) and therefore not included in the analysis; zero values (on the y axis) are cells with spontaneous spiking in control conditions <1 spike/s. (R and S) same plots as in G and K, but during whisking. (More details can be found in Figure S1).

Before beginning the optogenetic experiments, we first conducted electrophysiological recordings to verify that neuronal activity differs in the presence or absence of whisking, and to examine the effect of unexpected whisker deflection on this activity. Scrutiny of single-unit activity in S1L6, VPM and POm during phases of free whisking in awake, habituated, head-restrained mice revealed an increase in activity in all structures that was significantly less when whisking was absent (Figure 1A).

Analysis of single-unit activity revealed that in S1L6, random spontaneous spiking in the absence of whisking was significantly lower compared to when the animal was whisking (*p* = 0.0459, two-way analysis of variance with repeated measures (rmANOVA), *n* = 201; Tables 1 and S1) (Figure 1A). To mimic a sudden, deviant whisker stimulation event, an air puff was used to deflect the whiskers (whisker-evoked response, WER, 25 deflections each with 20 ms duration, repeated at 0.5 Hz). Here, single-unit activity in S1L6 increased roughly 20-fold (Table 1) whereas responses were significantly larger when WER occurred during whisking (*p* < 0.0001, two-way rmANOVA, *n* = 201; further details in Table S1).

**Table 1:** Summary of cortical and thalamic single-unit activity during whisking and non-whisking states

	S1 L6 spikes/s (*n* = 201)	VPM spikes/s (*n* = 63)	POm spikes/s (*n* = 155)
non- whisking	1.67 ± 0.26	5.47 ± 0.74	6.12 ± 0.44
whisking	5.60 ± 0.50	12.25 ± 1.12	11.42 ± 0.74
non- whisking + WER	22.53 ± 1.84	25.80 ± 1.99	31.35 ± 1.96
whisking +WER	31.73 ± 2.34	47.23 ± 4.04	38.38 ± 2.01

**Statistics Summary**			

non- whisking vs. whisking	*p* = 0.0459	*p* = 0.0075	*p* = 0.0004
non- whisking + WER vs. whisking + WER	*p* < 0.0001	*p* < 0.0001	*p* < 0.0001
L6 vs. POm (non- whisking)	*p* < 0.0001		
L6 vs. POm (whisking)	*p* < 0.0001		
L6 vs. VPM (non- whisking)	*p* < 0.0001		
L6 vs. VPM (whisking)	*p* < 0.0001		
POm vs. VPM (non- whisking)	*p* > 0.9999		
POm vs. VPM (whisking)	*p* > 0.9999		
L6 vs. POm (non- whisking +WER)	*p* < 0.0001		
L6 vs. POm (whisking+WER)	*p* = 0.0488		
L6 vs. VPM (non- whisking +WER)	*p* = 0.7408		
L6 vs. VPM (whisking+WER)	*p* < 0.0001		
POm vs. VPM (non- whisking +WER)	*p* = 0.1612		
POm vs. VPM (whisking+WER)	*p* = 0.0383		

The table summarizes the spontaneous single-unit activity in the presence or absence of external whisker deflections during whisking or non-whisking episodes. Comparisons were made between the cortical activity in S1L6 and thalamic activity. *p*-values report outcomes from two-way rmANOVA or one-way rmANOVA (see details in the main text and Supplementary file). Data are mean ± SEM. WER: whisker-evoked response.

With regard to POm, single-unit activity in the presence and absence of whisking was significantly greater than responses detected in S1L6 (Table 1) (*p* < 0.0001, one-way ANOVA). POm also showed a significant increase in spontaneous activity when non-whisking and whisking phases were compared (*p* = 0.0004; two-way rmANOVA, *n* = 155; Table 1; further details in Table S1), consistent with the findings of others.^39^ Single-unit activity during whisking and WER was significantly (roughly 5-fold) greater than WER in the absence of whisking (*p* < 0.0001; two-way rmANOVA, *n* = 155; Table 1). Single unit-activity during non-whisking and whisking, and WER was significantly greater than in S1 (p_non-whisking_ < 0.0001; p_whisking_ 0.0488; one-way ANOVA).

In VPM, single-unit activity was also significantly increased when activity was assessed during whisking compared to non-whisking states (*p* = 0.0075, two-way rmANOVA repeated on both factors, *n* = 63; Tables 1 and S1). Similar effects have been reported by others.^39^ Responses were not statistically significant from POm responses under the same conditions (*p* > 0.9999; one-way ANOVA) but were significant from S1L6 responses (*p* < 0.0001; one-way ANOVA). Single unit-activity increased roughly 5-fold when WER occurred during whisking compared to WER in non-whisking phases (*p* < 0.0001, two-way rmANOVA, *n* = 63; Table 1). Overall, POm and VPM exhibited significantly higher single unit activity compared to S1L6 during non-whisking and whisking phases (Table 1). Whereas WER during non-whisking was equivalent in S1L6 and VPM (*p* = 0.7408; one-way ANOVA), POm exhibited significantly stronger responses (Table 1).

These data indicate that S1 L6 exhibits much lower single unit activity compared to VPM and POm during non-whisking phases (S1L6 < POm = VPM), and all structures show a roughly 2-fold activity increase during whisking (S1L6 = POm = VPM). In addition, POm reacts more sensitively to WER during non-whisking phases (POm > VPM = S1L6) (*p* < 0.0001; comparison for linear trend in one-way ANOVA). Furthermore, although all structures showed response increases when WER occurred during whisking, VPM showed the most prominent increase (VPM > POm > S1) (*p* = 0.0163; comparison for linear trend in one-way ANOVA). These results raise the question as to whether corticothalamic modulation can be differentiated based on the inputs of S1L6 to POm and VPM, and whether this scrutiny can reveal different functions of these somatosensory thalamic structures.

### Inactivation of dopamine D1-expressing cells in S1 layer 6 silences activity in medial posterior and layer 6 during both whisking and non-whisking phases

To assess corticothalamic control of POm and VPM, Drd1-cre or Ntsr1-cre mice underwent the transfection of Archaerhodopsin (Arch) into S1L6 (Figures 1B–1F). Our strategy was based on the findings by others that an excitatory layer 6B projection from S1 to POm emanates from cells that express Drd1.^34^ Another excitatory cell population in layer 6, that expresses Ntsr1, projects to both VPM and POm.^36^ Application of green light (550 nm) was used to stimulate Arch in S1L6, and thus, inhibit cells that express Drd1 or Ntsr1. Electrophysiological recordings were conducted in the cortical layers of S1, as well as in POm or VPM, during the photoinactivation of either Drd1 or Ntsr1-expressing cells in layer 6. By this means, we aimed to discriminate the effect of these two different corticothalamic projections from S1 on neuronal responses in POm and VPM.

We then examined single-unit activity in S1 layer 6 and POm during non-whisking phases, when S1L6 Drd1-expressing cells were subjected to photoinactivation for 70 ms. Activity was significantly suppressed in both S1L6 Drd1-cells (from 4.87 ± 0.70 spikes/s to 1.75 ± 0.31 spikes/s; *n* = 46, Figures 1G–1I) and POm (from 7.09 ± 0.50 spikes/s to 3.79 ± 0.43 spikes/s; *n* = 98, Figures 1K and 1L) compared to controls during non-whisking phases (p_Drd1_ = 0.0002; p_POm_ < 0.0001; one-way rmANOVA; for further details see Table S2).

Upon the cessation of Drd1 cell inhibition, responses in POm recovered almost immediately, showing a transient increase of activity compared to baseline responses (from 7.09 ± 0.50 spikes/s in controls to 13.29 ± 1.42 spikes/s; *p* < 0.0001; one-way rmANOVA), that stabilized back to baseline levels by 100 msec after light application had ended (Figure 1K).

The assessment of photoinactivation (for 70 ms) of Drd1-expressing cells in S1L 6 on responses in other layers of S1 revealed no change in single-unit activity, when responses during non-whisking phases were compared in the presence or absence of Arch-mediated inhibition (Figures 1M–1Q) (Table S2). Moreover, cells in layer 6 that were classified as non-Drd1 cells (see STAR Methods) did not exhibit a change of activity during light application (Figure 1N). Furthermore, the activity of interneurons (*n* = 39) did not change under these circumstances in any of the cortical layers (Table S3). Thus, the effects we detected were restricted to S1L6.

It was recently reported that the activation of S1L6B activates POm, which subsequently activates S1L5.^40^ We did not see this during the photoinactivation of Drd1-expressing cells in S1L6B, using a light pulse of 70 ms duration. To clarify if we could, however, confirm the cortico-thalamo-cortical loop reported by Zolnik et al.,^39^ we extended the duration of the light pulse to 120 ms. Here, the suppression of single-unit activity in S1L6 and POm was similar to responses that occurred during light application for 70 ms (Figure S1). However, a significant decrease in activity was detected in S1L5 (Figure S1 and Table S2), consistent with the findings of Zolnik et al., as well as a suppression of activity in non-Drd1-expressing cells of layer 6 (Table S2), presumably due to rebound effects resulting from alterations in POm activity during the photoinactivation of S1L6.^41^ To exclude an influence of S1L5 and non-Drd1-expressing S1L6 cells in our scrutiny of the S1L6 projections to POm, all subsequent assessments were conducted using the photo-inactivation of 70 ms duration.

We then examined single-unit activity in S1L6 and POm during whisking phases, when S1L6 Drd1-expressing cells were photoinactivated for 70 ms. Activity was significantly suppressed in both S1L6 Drd1-cells (from 8.18 ± 1.27 spikes/s to 3.99 ± 0.76 spikes/s; *n* = 46, Figure 1R) and POm (from 14.67 ± 1.19 spikes/s to 7.66 ± 0.77 spikes/s; *n* = 98, Figure 1S), compared to controls during non-whisking phases (p_Drd1_ = 0.0001; p_POm_ < 0.0001; one-way rmANOVA). Interestingly the absolute decrease of the spontaneous activity in POm during S1L6-Drd1 inactivation was larger during whisking (absolute decrease POm non-whisking: 4.03 ± 0.26 Spikes/s; absolute decrease POm whisking: 7.01 ± 0.71 Spikes/s; *p* < 0.0001; paired Student’s *t* test, *t*(45) = 1.226), whereas the absolute decrease in L6 was similar (absolute decrease L6 non-whisking: 3.22 ± 0.35 Spikes/s; absolute decrease L6 whisking: 4.20 ± 0.69 Spikes/s; *p* = 0.2264; paired *t* test, *t*(97) = 4.129)).

These findings support that a corticothalamic projection to POm derives from Drd1-expressing cells of S1L6. Furthermore, these results show that the photoinactivation of Drd1-expressing cells in S1L6 not only reduces single-unit activity of Drd1-expressing cells in this cortical layer, but also significantly suppresses activity in POm, regardless of whether whisking is taking place or not.

### Inactivation of neurotensin receptor 1-expressing cells in S1 layer 6 silences activity in medial posterior, ventral posterior medial, and layer 6 during both non-whisking and whisking phases

**Figure 2:**
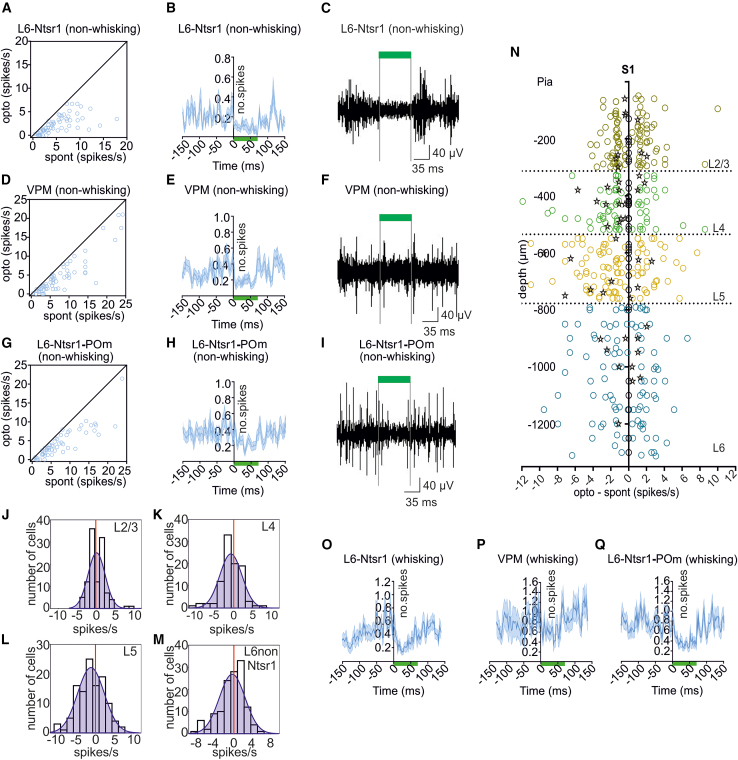
Inactivation of Arch-transfected S1L6-Ntsr1 cells with light pulses (70 ms, 550 nm, 35.83 mW/mm2) and the effect on spontaneous activity (A) Cells classified as L6-Ntsr1 (*n* = 64). The spontaneous activity, in the absence of a light pulse (“spont”) is plotted against spiking activity during the light pulse (“opto). (B) The averaged PSTHs (average in dark blue with light blue SEM confidence bands) of all 64 L6-Ntsr1 cells during inhibition with a 70 ms long light pulse (green bar) starting at time zero. (C) A representative *in vivo* electrophysiological recording of an L6-Ntsr1 cell during the light pulse application. (D) Same plot as (A) for VPM. (E) The averaged PSTHs of all 63 VPM cells during light pulse application. (F) A representative *in vivo* electrophysiological recording of a VPM cell. (G) Same plot as (A) for POm. (H) The averaged PSTHs of all 58 POm cells during light pulse application. (I) A representative *in vivo* electrophysiological recording from a POm cell. (J–N) The effect of L6-Ntsr1 photoinactivation on the spontaneous activity in the other cortical layers. (J–M) Histogram of all recorded cells/cortical layers. X axis: “opto” minus “spont,” calculated in a 70 ms time window. (N) Each cell from J–M is plotted separately at the recorded depth (y axis); x axis: “Opto” minus “Spont”; interneurons are indicated with stars; zero values (on the y axis) are cells with spontaneous spiking in control conditions (no light pulse) < 1 spike/s. (O–Q) same plots as in B, E, and H, but during whisking. (More details are available in Figure S2).

**Figure 3:**
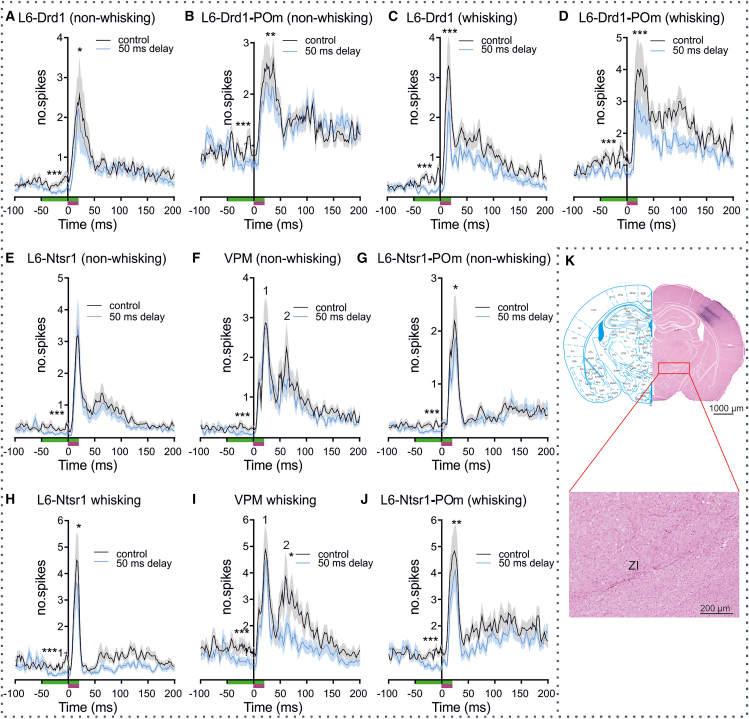
Effect of S1L6-Drd1 and L6-Ntsr1 inactivation on whisker-evoked responses (WER) occurring during whisking or non-whisking episodes (A) The averaged PSTHs (average with SEM confidence bands) of all 46 recorded S1L6-Drd1 cells shows spontaneous and whisker-evoked spiking activity during non-whisking. (B) Same plot as (A), but for all recorded POm cells (*n* = 98). (C) Same plot as (A) but: The averaged PSTHs of all 46 recorded S1L6-Drd1 cells during whisking. (D) Same plot as (C), but for all recorded POm cells. (E) The averaged PSTHs (average with SEM confidence bands) of all 64 recorded S1L6-Ntsr1 cells shows spontaneous and whisker-evoked spiking activity during non-whisking phases. (F) Same plot as (E), but for all 63 recorded VPM cells. (G) Same plot as (E), but for all 58 recorded POm cells. (H) Same plot format as for (E), but, panel shows the averaged PSTHs of all 64 recorded S1L6-Ntsr1 cells during whisking. (I) Same plot format as for (H), but for VPM. (J) Same plot as (H), but for POm. (The black curves show the control (without photoinactivation) and the blue curves the results with photoinactivation. The green bar shows the duration of S1L6-Drd1 or S1L6-Ntsr1 inactivation, which started 50 ms before the whisker deflection, and was conducted with a 20 ms air-puff (violet bar). The inactivation ended at the same time as the whisker deflection; stars represent significantly differences between the control (without light) and opto condition; the light intensity was 250 mA (35.83 mW/mm2)). (See also Figures S3 and S4). (K) Immuno-staining of mCherry-DAB-Ni in Drd1-cre mice. Floxed-mCherry was injected in S1L6, and an antibody-staining was performed to increase the fluorescence signal with DAB-Ni. The staining shows projection targets (black punctae) of S1L6-Drd1 cells in POm as well as zona incerta (ZI; zoomed image) (see also Figure S6).

We implemented a similar strategy, as above, to assess the effect of the photoinactivation of Ntsr1-expressing cells in S1L6 during whisking and non-whisking events. Responses were significantly suppressed during the photoinactivation of S1L6 for 70 ms in the absence of whisking, compared to non-whisking responses without photoinactivation, in S1L6 Ntsr1 cells (from 2.32 ± 0.26 spikes/s to 0.93 ± 0.14 spikes/s; *n* = 64, Figure 2A), VPM (from 5.55 ± 0.80 spikes/s to 3.18 ± 0.60 spikes/s; *n* = 63, Figure 3D) and POm (from 6.54 ± 0.81 spikes/s to 3.95 ± 0.57 spikes/s; *n* = 58, Figure 2G) (p_L6-Ntsr1_ < 0.0001; p_POm_ < 0.0001; p_VPM_ = 0.0025; one-way rmANOVA; Table S4). Responses in all three regions recovered rapidly after the cessation of the photoinactivation of Ntsr1-expressing cells of S1 L6 (Figures 2A–2I and Table S4).

We then examined single-unit activity in S1 layer 6, VPM, and POm during whisking phases, when S1L6 Ntsr1-expressing cells were photoinactivated for 70 ms. Responses in S1L6 Ntsr1 cells (from 7.40 ± 0.72 spikes/s to 3.67 ± 0.43 spikes/s; *n* = 64, Figure 2O), VPM (from 13.83 ± 1.51 spikes/s to 8.35 ± 0.93 spikes/s; *n* = 63, Figure 2P) and POm (from 12.68 ± 1.16 spikes/s to 6.62 ± 0.63 spikes/s; *n* = 58, Figure 2Q) were significantly suppressed during the photoinactivation of S1L6 in the absence of whisking, compared to non-whisking responses in baseline conditions(p_L6-Ntsr1_ < 0.0001; p_POm_ < 0.0001; p_VPM_ < 0.0001; one-way rmANOVA). Interestingly the absolute decrease of the spontaneous activity in POm and VPM during S1L6-Ntsr1 inactivation was also larger during whisking (absolute decrease POm non-whisking: 4.00 ± 0.58 Spikes/s; absolute decrease POm whisking: 6.16 ± 0.70 Spikes/s; *p* = 0.0260; paired *t* test, *t*(57) = 2.286; absolute decrease VPM non-whisking: 3.12 ± 0.32 Spikes/s; absolute decrease VPM whisking: 5.47 ± 0.84 Spikes/s; *p* = 0.0142; paired *t* test, *t*(62) = 2.524). By contrast, the absolute decrease in L6 was similar (absolute decrease L6 non-whisking: 2.92 ± 0.35 Spikes/s; absolute decrease L6 whisking: 3.72 ± 0.45 Spikes/s; *p* = 0.01938; paired *t* test, *t*(63) = 1.314)).

There was no effect of the photoinactivation of S1L6 Ntsr1-expressing cells on spontaneous spiking in the other cortical layers, or in non-Ntsr1 cells of S1L6 (Figures 1J–2N and S2) (one-way ANOVA with repeated measures, *p* > 0.08; L2/3 *n* = 101, L4 *n* = 82, L6 non-Ntsr1 *n* = 118; *N* = 12 animals; Table S4). Moreover, the activity of interneurons (*n* = 46) did not change under these circumstances (Table S5). In summary, these data support that a corticothalamic projection originating in Ntsr1-expressing neurons of S1L6 modulates the activity of neurons within *both* POm and VPM.

### Externally triggered whisker deflection response in S1 layer 6 and medial posterior during whisking and non-whisking events is suppressed by the inactivation of dopamine D1-expressing cells

Having found that the photoinactivation of Drd1-expressing cells in S1L6 reduces single-unit activity in both S1L6 *and* POm during non-whisking phases, we now addressed the extent to which this corticothalamic response is altered when whisker deflection is externally implemented. Controls experienced 25 air-puffs of 20 ms duration at a frequency of 0.5 Hz. We then assessed the whisker-evoked response (WER) during active whisking and non-whisking events.

WER revealed a highly significant increase in single-unit activity; during non-whisking and whisking events, in both S1 L6 (Figures 3A and 3C; Table 1) and POm (Figures 3B and 3D and Table 1). Photoinactivation of S1L6 was started 50 ms before whisker deflection. This delay ensured that the photoinactivation of S1L6 Drd1-expressing cells had enough time to affect neuronal responses in the thalamus and the other cortical layers, before the stimuli originating in the peripheral whisker input reached the thalamus and the cortical layers. Photoinactivation resulted in a significant suppression of single-unit responses in S1L6 Drd1-expressing cells (Figures 3A and 3C) and in POm (Figures 3B and 3D), during phases of active whisking and non-whisking (two-way ANOVA repeated on both factors; L6-Drd1 WER during non-whisking decreased from 41.68 ± 4.74 spikes/s to 36.38 ± 4.42 spikes/s, *p* < 0.0001; L6-Drd1 WER during whisking decreased from 37.70 ± 4.01 spikes/s to 26.80 ± 3.54 spikes/s, *p* < 0.0001; POm WER during non-whisking decreased from 31.76 ± 2.79 spikes/s to 27.31 ± 2.52 spikes/s, *p* < 0.0001; POm WER during whisking decreased from 38.84 ± 2.73 spikes/s to 30.11 ± 2.09 spikes/s, *p* < 0.0001; statistics in Table 2). Furthermore, when applied during free-whisking, WER in S1L5 decreased during the photoinactivation of S1L6-Drd1 cells compared with control conditions (from 37.83 ± 3.31 to 33.29 ± 3.26 spikes/s, *p* = 0.0004; statistics in Table 2) (Figure S3). Although responses were significantly suppressed under these conditions, they were not abolished. These findings suggest that the S1L6 Drd1-POm projection may support the detection of deviant or salient tactile stimuli.

**Table 2:** Effect of photoinactivation of S1L6-Drd1 cells on whisker-evoked activity during whisking and non-whisking states

	L2/3	L4	L5	L6 non-Drd1	L6-Drd1	POm
	spikes/s (non-whisking)					
spont. ctrl.	4.24 ± 0.81	2.40 ± 0.56	1.80 ± 0.56	1.32 ± 0.38	5.76 ± 1.29	5.71 ± 0.52
spont. opto	4.21 ± 0.80	2.16 ± 0.58	1.57 ± 0.36	1.15 ± 0.33	2.61 ± 0.87	2.60 ± 0.37
WER ctrl.	21.38 ± 2.33	34.70 ± 3.72	42.67 ± 3.44	33.15 ± 2.43	41.68 ± 4.74	31.76 ± 2.79
WER+opto	22.64 ± 2.64	32.95 ± 3.32	40.26 ± 3.30	31.53 ± 2.46	36.38 ± 4.42	27.31 ± 2.52
two-way ANOVA	*p* value					
spont-WER	<0.0001 F(1,62) = 63.31	<0.0001 F(1,59) = 89.74	<0.0001 F(1,66) = 156.8	<0.0001 F(1,75) = 170.9	<0.0001 F(1,45) = 71.89	<0.0001 F(1,97) = 100.4
ctrl.vs. opto	0.5490 F(2,124) = 0.6027	0.3611 F(2,118) = 1.028	0.2298 F(2,132) = 1.487	0.2054 F(2,150) = 1.600	<0.0001 F(2,90) = 11.42	<0.0001 F(2,194) = 24.18
Interaction	0.3649 F(2,124) = 1.016	0.6397 F(2,118) = 0.448	0.5474 F(2,132) = 0.6054	0.2623 F(2,150) = 1.350	0.4508 F(2,90) = 0.8039	0.4354 F(2,194) = 0.8350
Post-hoc test	*p* value					
Spont. ctrl. vs. opto	>0.9999 t(124) = 0.03570	>0.9999 t(118) = 0.2102	>0.9999 t(132) = 0.1426	>0.9999 t(150) = 0.2262	0.0254 t(90) = 2.543	0.0003 t(194) = 3.865
WER ctrl. vs. opto	0.2289 t(124) = 1.590	0.2495 t(118) = 1.546	0.2779 t(132) = 1.489	0.0722 t(75) = 2.115	<0.0001 t(90) = 4.273	<0.0001 t(194) = 5.520
	spikes/s (whisking)					
spont. ctrl.	2.54 ± 0.52	4.40 ± 0.77	5.39 ± 7.37	2.28 ± 0.51	8.92 ± 1.32	12.71 ± 1.02
spont. opto	3.01 ± 0.60	4.14 ± 0.78	3.77 ± 5.39	2.49 ± 0.67	5.00 ± 0.99	7.88 ± 0.72
WER ctrl.	21.49 ± 2.56	32.18 ± 3.02	37.83 ± 3.31	27.92 ± 2.27	37.70 ± 4.01	38.80 ± 2.73
WER+opto	21.72 ± 2.48	31.87 ± 3.12	33.29 ± 3.26	25.38 ± 2.24	26.80 ± 3.54	30.11 ± 2.09
two-way ANOVA	*p* value					
spont-WER	<0.0001 F(1,62) = 64.20	<0.0001 F(1,59) = 102.1	<0.0001 F(1,66) = 96.23	<0.0001 F(1,75) = 152.2	<0.0001 F(1,45) = 63.91	<0.0001 F(1,97) = 140.6
ctrl. vs. opto	0.3262 F(2,124) = 1.131	0.6879 F(2,118) = 0.3753	0.0006 F(2,132) = 7.888	0.3147 F(2,150) = 1.165	<0.0001 F(2,90) = 26.96	<0.0001 F(2,194) = 57.71
Interaction	0.9606 F(2,124) = 0.0402	0.1469 F(2,118) = 1.949	0.2102 F(2,132) = 1.578	0.2294 F(2,150) = 1.487	<0.0001 F(2,90) = 12.22	0.0006 F(2,194) = 7.794
Post-hoc test	*p* value					
Spont. ctrl. vs. opto	>0.9999 t(124) = 0.6686	>0.9999 t(118) = 0.2093	0.3430 t(132) = 1.375	>0.9999 t(150) = 0.1824	0.0009 t(90) = 3.640	<0.0001 t(194) = 5.608
WER ctrl. vs. opto	>0.9999 t(124) = 0.3294	>0.9999 t(118) = 0.2423	0.0004 t(132) = 3.859	0.0623 t(150) = 2.175	<0.0001 t(90) = 10.13	<0.0001 t(194) = 10.15

The table shows the spiking activity (spikes/s) for each cortical layer, S1L6-Drd1, and POm cells during spontaneous activity (spont.), whisker-related spont. activity and whisker-evoked responses (WER) with (opto, light intensity 250 mA) and without optogenetic (ctrl.) inactivation of S1L6-Drd1 cells: (L2/3 *n* = 63, L4 *n* = 60, L5 *n* = 67, L6 non-Drd1 *n* = 76 (3 cells, same as in Table S2, excluded), S1L6-Drd1 *n* = 46, POm *n* = 98 (10 animals)). Two-way ANOVA repeated on both factors (row factor: spont. vs. WER; column factor: ctrl vs. opto) with Bonferroni post-hoc tests done separately on data from each cortical layer, S1L6-Drd1 and POm. Data represented SUA and reported as mean ± SEM. (Data represented in Figures 3 and S3).

### Inactivation of neurotensin receptor 1-expressing cells during externally triggered whisker deflection reveals a state-dependent contribution of ventral posterior medial and medial posterior to this corticothalamic response

Next, we assessed the effect of WER during active whisking and non-whisking episodes in Ntsr1-Cre mice. For this, we assessed responses in S1L6, POm, and VPM.

WER triggered single-unit activity in Ntsr1-expressing cells of S1L6 (Figures 3E and 3H), in VPM (Figures 3F and 3I), and POm (Figures 3G and 3J) during non-whisking and whisking phases (Table 3). The WER response in POm occurred from ∼6 ms to 38 ms after whisker stimulation onset. WER activity in VPM was biphasic, consistent with the findings of others.^42^^,^^43^ The early response occurred from ∼5 ms until 40 ms after stimulus onset, and the late response occurred from ∼41 ms until 100 ms after stimulus onset. Photo-inactivation of S1L6 Ntsr1 cells beginning 50 ms before WER during non-whisking episodes did not alter single-unit responses in S1L6 (Figure 3E), or VPM (Figure 3F), but significantly diminished responses in POm (Figure 3G) (from 30.66 ± 2.27 spikes/s to 27.37 ± 2.41 spikes/s; *p* = 0.0144, two-way ANOVA repeated on both factors, exact numbers and statistics in Table 3). Activity in L2/3 of S1 was also decreased (from 36.03 ± 3.36 to 33.25 ± 3.36, *p* = 0.0273; statistics in Table 3) (Figure S4).

**Table 3:** Effect of photoinactivation of S1 L6-Ntsr1 cells on whisker-evoked activity during whisking and non-whisking states

	L2/3	L4	L5	L6 non-Ntsr1	L6-Ntsr1	VPM	POm
	spikes/s (non-whisking)						
spont. ctrl.	3.15 ± 0.60	4.54 ± 0.74	4.59 ± 0.61	2.40 ± 0.42	4.73 ± 0.55	5.47 ± 0.74	7.27 ± 0.86
spont. opto	3.36 ± 0.60	3.79 ± 0.76	3.78 ± 0.53	2.02 ± 0.37	1.29 ± 0.17	2.15 ± 0.33	3.31 ± 0.50
WER ctrl.	23.23 ± 2.57	39.11 ± 2.64	43.13 ± 2.81	36.55 ± 2.83	31.11 ± 2.95	25.80 ± 1.99	30.66 ± 2.27
WER+opto	25.65 ± 3.17	42.31 ± 3.64	42.45 ± 3.00	36.67 ± 3.03	31.08 ± 3.10	25.81 ± 1.90	27.37 ± 2.41
WER2 ctrl.	X	X	X	x	x	20.30 ± 2.59	x
WER2+opto	X	X	X	x	x	18.15 ± 1.86	x
two-way ANOVA	*p* value						
spont-WER	<0.0001 F(1,100) = 58.13	<0.0001 F(1,82) = 153.0	<0.0001 F(1,103) = 211.1	<0.0001 F(1,118) = 156.5	<0.0001 F(1,63) = 115.2	<0.0001 F(1,62) = 184.4	<0.0001 F(1,57) = 159.8
ctrl. vs. opto	0.0029 F(2,200) = 6.03	0.0341 F(2,164) = 3.45	0.6377 F(2,206) = 0.45	0.0687 F(2,236) = 2.71	0.1832 F(2,126) = 1.72	0.0419 F(2,124) = 3.26	0.0007 F(2,114) = 7.74
Interaction	0.0491 F(2,200) = 3.060	0.0844 F(2,164) = 2.510	0.9189 F(2,206) = 0.08460	0.0955 F(2,236) = 2.372	0.1394 F(2,126) = 2.002	0.1427 F(2,124) = 1.978	0.3398 F(2,114) = 1.089
post-hoc test	*p* value						
spont. ctrl. vs. opto	>0.9999 t(200) = 0.2259	>0.9999 t(164) = 0.487	>0.9999 t(206) = 0.6709	>0.9999 t(236) = 0.3926	0.0337 t(126) = 2.423	0.0117 t(124) = 2.805	0.0026 t(114) = 3.295
WER ctrl. vs. opto	0.0149 t(200) = 2.703	0.0760 t(164) = 2.092	>0.9999 t(206) = 0.5667	>0.9999 t(236) = 0.120	>0.9999 t(126) = 0.0202	>0.9999 t(124) = 0.0068	0.0144 t(114) = 2.736
WER2 ctrl. vs. opto	X	X	X	x	x	0.9999 t(124) = 0.3452	x
	spikes/s (whisking)						
spont. ctrl.	6.09 ± 0.94	6.82 ± 0.86	6.31 ± 0.61	4.65 ± 0.57	8.02 ± 0.88	12.25 ± 1.12	9.38 ± 0.92
spont. opto	5.46 ± 0.76	7.60 ± 0.93	6.26 ± 0.60	5.68 ± 0.65	4.18 ± 0.64	7.48 ± 0.87	4.76 ± 0.51
WER ctrl.	36.03 ± 3.36	49.90 ± 3.47	51.02 ± 3.39	46.17 ± 3.16	48.83 ± 4.42	47.23 ± 4.04	37.84 ± 2.76
WER+ opto	33.25 ± 3.36	51.18 ± 3.35	49.91 ± 3.22	43.03 ± 2.95	42.46 ± 3.99	43.49 ± 3.69	30.70 ± 2.58
WER2. ctrl.	X	X	x	x	x	33.29 ± 1.12	x
WER2+opto	X	X	x	x	x	25.00 ± 3.29	x
One-way ANOVA	*p* value						
spont-WER	<0.0001 F(1,100) = 98.5	<0.0001 F(1,82) = 200.6	<0.0001 F(1,103) = 219	<0.0001 F(1,118) = 208	<0.0001 F(1,63) = 111.3	<0.0001 F(1,62) = 156.1	<0.0001 F(1,57) = 151
ctrl. vs. opto	0.0491 F(2,200) = 3.059	0.5245 F(2,164) = 0.6478	0.6813 F(2,206) = 0.3844	0.4227 F(2,236) = 0.4227	0.0002 F(2,126) = 8.970	0.0140 F(2,124) = 4.419	<0.0001 F(2,114) = 23.73
Interaction	0.3737 F(2,200) = 1.0	0.4664 F(2,164) = 0.76	0.1987 F(2,206) = 1.63	0.0153 F(2,236) = 4.26	0.4955 F(2,126) = 0.7	0.0351 F(2,124) = 3.44	0.1615 F(2,114) = 1.8
post-hoc test	*p* value						
spont. ctrl. vs. opto	>0.9999 t(200) = 0.561	>0.9999 t(164) = 0.587	>0.9999 t(206) = 0.0414	0.5961 t(236) = 1.043	0.0369 t(126) = 2.387	0.0362 t(124) = 2.395	<0.0001 t(114) = 4.287
WER ctrl. vs. opto	0.0273 t(200) = 2.489	0.7350 t(164) = 0.920	0.8392 t(206) = 0.8088	0.0054 t(236) = 3.031	0.0002 t(126) = 3.969	0.1249 t(124) = 1.880	<0.0001 t(114) = 6.620
WER2 ctrl. vs. opto	X	X	x	x	x	0.0303 t(124) = 2.516	x

The table shows spiking activity (spikes/s) for each cortical layer, as well as S1L6-Ntsr1, VPM, and POm cells during spontaneous activity (spont.), whisker-related spont. activity and whisker-evoked responses (WER) (light pulse 70 ms, 550 nm, intensity 250 mA) and without optogenetic (ctrl.) photoinactivation of S1L6-Ntsr1 cells: (L2/3 *n* = 101, L4 *n* = 83, L5 *n* = 104, L6 non-Ntsr1 *n* = 119, L6-Ntsr1 *n* = 64, VPM *n* = 63, POm *n* = 58 (12 animals)). Two-way ANOVA repeated on both factors (row factor: spont. vs. WER; column factor: ctrl vs. opto) with Bonferroni post-hoc tests done separately on data from each cortical layer, S1L6-Ntsr1, VPM, and POm. Data are SUA and reported as mean ± SEM. (Date represented in Figures 3 and S4).

By contrast, photo-inactivation of S1L6 Ntsr1 cells, during ongoing active whisking, significantly reduced the WER effect in S1L6 (Figure 3H) (from 48.83 ± 4.42 spikes/s to 42.46 ± 3.99 spikes/s; *p* = 0.0002) and POm (Figure 3J) (from 37.84 ± 2.76 spikes/s to 30.70 ± 2.56 spikes/s; *p* < 0.0001), and also significantly suppressed the second peak of the VPM response (Figure 3I) (from 33.29 ± 4.17 spikes/s to 25.00 ± 3.29 spikes/s; *p* = 0.0303), (two-way ANOVA repeated on both factors, exact numbers and statistics in Table 3). These findings suggest that the engagement of Ntsr1-expressing cells of S1L6 and their respective projections to VPM by a deviant whisking event is salient only during whisking, whereas the S1L6 Ntsr1-related projection to POm reacts in a state-independent manner to this kind of stimulus.

### S1 layer 6-dopamine D1 inactivation has a larger impact on the whisker-evoked responses in medial posterior than does S1 layer 6-neurotensin receptor 1 inactivation

To further investigate the difference between L6-Drd1 and L6-Ntsr1 cell modulation of POm activity, the effect of the photoinactivation of these cell populations on WER was compared, when the spiking activity *preceding* light stimulation was subtracted. WER during non-whisking phases was similar during S1L6-Drd1 and S1L6-Ntsr1 cell photoinactivation (WER-POm_Drd1_: 25.00 ± 24.47 spikes/s, *n* = 98; *N* = 10 animals; WER-POm_Ntsr1_: 24.06 ± 17.58 spikes/s, *n* = 58; *N* = 12 animals; unpaired *t* test, *p* = 0.7978; *t*(155) = 0.2566). This suggests that both S1L6-Drd1 and S1L6-Ntsr1 cells contribute to POm excitation to a similar degree in this behavioral context. Interestingly, during whisking, the inactivation of S1L6-Drd1 cells had a *larger* effect on WER compared to the inactivation of S1 L6-Ntsr1 cells (WER-POm_Drd1_: 20.80 ± 12.87 spikes/s, *n* = 98; *N* = 10 animals; WER-POm_Ntsr1_: 25.94 ± 18.54 spikes/s, *n* = 58; *N* = 12 animals; unpaired *t* test, *p* = 0.0438; *t*(155) = 2.033). These differences in the absolute change of WER in POm, during non-whisking and whisking episodes, when the effects of S1L6-Drd1 and S1L6-Ntsr1 photoinactivation were compared, were further analyzed and confirmed with linear regression plots (Supplementary Info 1 and Figure S5).

We then examined the difference between spontaneous activity and WER to see if we could validate the abovementioned findings. The interpretation that the effect of photoinactivation is larger in one condition (whisking or non-whisking) would be validated if a significant interaction between the manipulation (photoinactivation versus control) and whisker stimulation (whisker-evoked activity versus spontaneous activity) is evident. Analysis revealed that S1L6-Drd1 photoinactivation has a greater impact on POm activity when the animal is engaged with whisking (Interaction factor POm_L6-Drd1_ non-whisking *p* = 0.4354 and whisking *p* = 0.0006; *n* = 98; Interaction factor of two-way rmANOVA). By contrast, S1L6-Ntsr1 cell photoinactivation influences POm equally during non-whisking and whisking (POm_L6-Ntsr1_ no whisking *p* = 0.3398 and whisking *p* = 0.1615; *n* = 58; Interaction factor of two-way rmANOVA). Thus, the abovementioned interpretation is valid.

### S1 layer 6 dopamine D1-expressing cells project to the zona incerta

A projection from zona incerta (ZI) to POm has been reported.^44^ Moreover, Bech et al. showed that L6-Ntsr1 cells project to ZI.^37^ We, thus, explored whether S1L6 Drd1-cells project to this structure. By means of DAB-Ni histological (i.e., 3,3′-diaminobenzidine with 1% nickel-ammonium-sulfate) analysis, we identified a novel projection from S1L6 Drd1-expressing cells to the ZI (Figures 3K and S6). This GABAergic structure,^45^ responds to whisker deflections during goal-directed behavior.^46^ We speculated that this function may relate to the detection of subtle whisker deflections during exploration,^47^ given that POm elicits a fast-onset, all-or-none response in S1L 2/3 in response to whisker deflection.^48^

We thus assessed the slope of WER detected in POm, during whisking and non-whisking phases in the presence and absence of photoinactivation of S1L6 cells (Figures 3B and 3D). We found that the WER slopes in POm (compared between control conditions and with L6-Drd1 or L6-Ntsr1 photoinactivation) were unchanged when whisker deflection occurred in the absence of whisking (POm_Drd1_ slope control 1.83 ± 0.26 spikes/s, slope opto 2.11 ± 0.30 spikes/s, *p* > 0.9999; one-way rmANOVA, *n* = 98; POm_Ntsr1_ slope control 1.26 ± 0.13 spikes/s, slope opto 1.36 ± 0.20 spikes/s, *p* > 0.9999; one-way rmANOVA, *n* = 58). By contrast, the slope of WER was significantly lower when responses in L6-Drd1 photoinactivated animals were compared with controls during whisking (POm_Drd1_ slope control 3.31 ± 0.38 spikes/s, slope opto 2.13 ± 0.27 spikes/s *p* = 0.0100; one-way rmANOVA, *n* = 98), whereas the slope of WER was unchanged when responses in L6-Ntsr1 photoinactivated animals were compared with controls during whisking (POm_Ntsr1_ slope control 2.84 ± 0.32 spikes/s, slope opto 2.36 ± 0.27 spikes/s, *p* > 0.9999; one-way rmANOVA, *n* = 58). Furthermore, when the slope of WER was compared in control animals during whisking and non-whisking phases, effects were significantly greater in the whisking condition (p_POm-Drd1_ = 0.0114, *n* = 98; p_POm-Ntsr1_ < 0.0001; one-way, *n* = 58; rmANOVA).

## Discussion

In this study, we assessed the functional relevance of two distinct corticofugal projections from S1L6 to the somatosensory thalamus. We scrutinized Drd1 and Ntsr1-expressing cells of S1L6 using an optogenetic and electrophysiological strategy. Inhibition of the activity of these cells for periods of 70 ms reduced neuronal activity of S1L6, without affecting the other cortical layers of S1. In line with previous reports of target-specific excitatory projections of S1L6 neurons to the somatosensory thalamus,^34^^,^^36^ we showed that the photoinactivation of Ntsr1-cell activity in S1L6 reduced neuronal activity in VPM and POm, whereas the photoinactivation of S1L6 Drd1-cell activity reduced activity in POm. These effects were evident while animals were engaged in whisking, or when their whiskers were quiescent, whereby the photoinactivation of both kinds of S1L6 cells affected POm more when the animal was whisking in the absence of whisker deflection. POm showed a more sensitive innate response to whisker deflection during non-whisking phases, but the photoinactivation of the S1L6 Drd1-pathway elicited a potent suppression of POm WER during whisking. Strikingly, photoinactivation of the S1L6 Ntsr1 cell-projection to VPM only modulated the whisker-evoked responses (WER) during active whisking (Table 4).

**Table 4:** Summary of statistically significant effects of inhibition of Ntsr1-expressing or Drd1-expressing cells of S1L6 in the primary somatosensory cortex during different somatosensory activity states

	Inactivation S1 L6 Ntsr1	Inactivation S1 L6 Drd1
POm non-whisking	↓	↓
VPM non-whisking	↓	–
S1 non-whisking	↓	↓
POm non-whisking + whisker deflection	↓	↓
VPM non-whisking + whisker deflection	N.E.	–
S1 non-whisking +whisker deflection	N.E.	↓
POM whisking	↓↓	↓↓
VPM whisking	↓	–
S1 whisking	↓	↓
POm whisking + whisker deflection	↓	↓↓
VPM whisking + whisker deflection	↓ 2ND peak	–
S1 whisking + whisker deflection	N.E	↓

Photoinactivation of Ntsr1-expressing, or Drd1-expressing, cells of S1L6 during electrophysiological recordings in awake mice revealed a significant suppression of neuronal activity in S1L6, POm, and VPM when the animals were not whisking (quiescent) or whisking. The absolute decrease of POm activity during S1L6 inhibition is larger during whisking. When whisker deflection was implemented while animals were quiescent, the whisker-evoked response (WER) was suppressed in POm, but not VPM or S1L6 during L6-Ntsr1-inhibition.

When whisker deflection was implemented during whisking, inactivation of S1L6 Ntsr1-expressing cells resulted in a suppression of WER in all three regions, whereby the second component of the biphasic VPM WER response was affected.

Inactivation of S1L6 Drd1-expressing cells also suppressed WER in POm and S1 L6, but the effect on POm was significantly greater than the same effect mediated by Ntsr1-cell inactivation.

Drd1-effects were only assessed in POm and S1L6, given that this cell population does not project to VPM.^34^

N.E.: no effect, single arrow signifies response suppression, double arrow signifies a significantly more potent effect of S1L6 Drd1-cell inhibition compared to S1L6 Ntsr1-cell inhibition in the condition indicated (see Tables 2 and 3 for statistical comparisons).

We observed that the corticothalamic projections of these cell populations to the somatosensory thalamus could be differentiated on the basis of intrinsic, behavioral, and externally generated ongoing somatosensory activity. Firstly, the influence of the S1L6-Drd1 cell projection on POm activity during whisking was stronger than the impact of the S1L6-Ntsr1 cell projection on POm (Table 4). Secondly, neuronal responses in POm, when whisker deflection was externally generated during the inhibition of S1L6 Ntsr1 or Drd1-expressing cells, were more potently suppressed when the deflection occurred during whisking. Thirdly, whisker deflection during the inhibition of S1L6 Ntsr1-expressing cells only exhibited a significant effect on VPM activity when the deflection occurred *during* whisking, whereas POm activity was affected under both whisking and non-whisking conditions. Strikingly, in the latter case, S1L6 responses were not altered when photoinactivation was implemented during WER in non-whisking phases. These findings point toward a role of the S1L6 Ntsr1-pathway to VPM in dynamic spatiotemporal item identification during whisking, whereas the S1L6 Ntsr1-and Drd1-pathways to POm may support the tuning of signal-to-noise ratios (Ntsr1 pathway) during externally initiated whisker deflections (Drd1 pathway).

Our research strategy was based on the findings by others that an excitatory layer 6B projection from S1 to POm emanates from cells that express Drd1.^34^ Another excitatory cell population in layer 6, that expresses the neurotensin receptor 1 (Ntsr1) projects to both VPM and POm.^36^ Thus, when exploring the role of corticothalamic S1L6 Drd1-cell projections to the somatosensory thalamus we recorded electophysiologically from POm. When assessing the influence of S1L6 Ntsr1-cell projections, we scrutinized responses in both structures. Given that S1L6 not only projects to the thalamus, but also projects to S1L 2/3,^36^^,^^37^ we also scrutinized the effect of the photo-inactivation of either Drd1-expressing or Ntsr1-expressing cells in S1L6 on activity in different cortical layers of S1, as well as the somatosensory thalamus. In general, we observed that photoinactivation of either pathway reduced single unit responses of POm during whisking and non-whisking phases, whereas the photoinactivation of the Ntsr1 pathway reduced activity of VPM.

### S1 layer 6, ventral posterior medial, and medial posterior exhibit different degrees of single-unit activity during behavioral states

Initially, we assessed single unit activity in S1L6, VPM, and POm during different somatosensory behavioral states. We found that S1L6 activity was significantly less active during whisking and non-whisking states, compared to VPM and POm. However, activity in S1L6 was similar to VPM when externally triggered whisker-responses occurred during non-whisking states. whereas POm activity was significantly higher compared to both regions under this condition. By contrast, when the externally triggered whisker-response occurred during whisking, the activity in VPM was significantly higher compared to S1L6 or POm.

These observations align with the proposals by others as to the function of VPM and POm: whereas POm may process experience-dependent whisker kinematics,^2^ VPM may support dynamic information processing about the temporal and discrete physical aspects of tactile features during ongoing exploration.^3^^,^^11^ A role for POm in stimulus-reward associations has also been proposed,^18^ but our experimental paradigm was not designed to address this property.

### Photoinactivation of S1 layer 6 dopamine D1 receptor-expressing cells suppresses S1 layer 6 and medial posterior activity regardless of behavioral state

POm has been proposed to support the integration of corticothalamic motor and tactile information^15^ thereby relaying information about whisker kinematics, and to help optimize tactile signal-to-noise ratios by helping to discriminate subthreshold from suprathreshold stimuli.^16^ In a previous study, where Drd1-cells of S1L6 were photoactivated by the light stimulation of channelrhodopsin^32^ it was reported that this S1L6-POm input triggered an initial increase in WER that declined while photoactivation was ongoing. Thus, if S1L6-Drd1 cell activation does *not* promote a sustained, repetitive activation of POm, such that its activity follows whisker movements, then perhaps the S1L6-Drd1 to POm connection is activated during specific circumstances.^49^ Our data suggest that POm is particularly involved in deviance detection, especially when this is unexpected: Of all regions assessed, it showed the greatest increase in single-unit activity when whiskers were deflected during non-whisking phases, although it also responded to WER during whisking. Photoinactivation of S1L6 Drd1-cells significantly reduced single unit responses in POm under these circumstances, whereby effects were more potent on POm activity during whisking, compared to non-whisking phases. This suggests that this specific corticothalamic input to POm may also support the detection of weak whisker deflections during active exploration,^47^ when head, and thus whisker readjustment becomes important for avoiding or detecting obstacles in space, as well as estimating the width of passageways. This also raises the interesting possibility that POm may be involved in the processing of vibrissa micro-motions that occur when rodents explore the texture of surfaces.^50^ In addition, the activity-dependent modulation, by the S1L6-Drd1 cell projection, of POm could be related to the differentiation of self-induced movement (i.e., expected whisker deflection) from a whisker deflection caused by an unexpected external source, as suggested by a strong interaction of S1L6 cells in the somatosensory and motor cortex with POm.^51^^,^^52^ In line with these interpretations, POm triggers a fast-onset, all-or-none response in S1L2/3^48^ that is consistent with its proposed involvement in suprathreshold deviant detection.

The abovementioned effects were most likely mediated by the direct input from S1L6-Drd1 cells to POm, given that single unit responses had a latency of <10 ms and WER onset was faster in POm than in S1L6. Although there is no evidence that S1L6-Drd1 cells project to the GABAergic reticular thalamic nucleus (TRN)^34^ that exerts an inhibitory control over the somatosensory relay nuclei, we identified a novel S1L6-Drd1 cell projection to the GABAergic zona incerta (ZI), an inhibitory subthalamic structure that influences thalamic information processing.^53^ We assessed the slope of WER and found no significant difference in responses in the absence of whisking, whereas effects were significant when WER occurred during ongoing whisking, when the photoinactivation of Drd1 cells was compared to controls. This S1L6- Drd1 pathway may therefore explain why the ZI responds to whisker deflections.^46^ Although these findings do not offer direct proof that ZI contributes to the detection of whisker deflection during whisking, they indicate that another physiological process is engaged to support the detection of whisker deflection during ongoing exploration. Taken together with the abovementioned reports that ZI responds to whisker deflections during goal-directed behavior,^46^ this places it as a candidate for the detection of by POm of subtle whisker deflections.

For example, the activation of ZI results in the suppression of activity in POm^54^ that may support stimulus selection. If the S1L6 input to ZI was reduced by the photoinactivation of Drd1-expressing cells, then one might have expected reduced inhibition of POm and thus an absence of response suppression in this structure. This was not the case under the conditions tested here, but may possibly be explained by the fact that VPM also projects to ZI.^55^ Whisker responsive neurons in ZI are reportedly few,^46^ albeit distributed with different densities along its rostrocaudal axis.^56^ However, short latency and precisely timed responses to whisker stimulation have been demonstrated in this structure.^57^ Thus, it is also possible that the effects of local photoinactivation in S1 layer 6 did not result in an appreciable reduction of activity in ZI that could be detected in POm. By contrast, when we compared the whisker evoked responses in POm in the presence and absence of whisking in control animals, the slope of the single unit response was steeper in the absence of whisking, suggesting that another physiological process might have been engaged in modulating the active whisking WER. The ZI is involved in goal-directed movement.^58^ The S1L6-Drd1 cells input to ZI may play a role in this property when it is related to whisking.

Interestingly, the activity of ZI is inhibited by the cholinergic laterodorsal tegmentum (LDT) and the pedunculopontine tegmentum (PPT), which are activated by increased arousal.^54^ This indicates that the ZI input might be more relevant during whisking than non-whisking states where the animal is not actively exploring (low arousal). It could, thus, be involved in deviant detection, because this stimulus is likely to arrive in POm before the cholinergic input is activated.

### Photoinactivation of neurotensin receptor 1-expressing cells in S1 layer 6 reveals a specific role for this pathway when whisker deflection occurs during whisking

The corticothalamic WER in VPM is characterized by two components^43^: the first component appears 3–10 ms, and the second appears 10–100 ms, after whisker deflection. Our data perfectly reflect this previously reported timelines, since we found that the early component appeared ∼5 ms after whisker stimulation and the late component ∼41 ms after whisker stimulation.

Whereas the first component is elicited by the relaying of whisker deflection along the leminiscal pathway,^2^^,^^3^ the origin of the second component is less clear and has been proposed to be mediated by excitation ramping up in the VPM.^43^

When Ntrsr1-expressing cells in S1L6 were photoinactivated during WER, an inhibition of VPM was evident during whisking, but *not* during non-whisking phases. Here, it was the *second* component of WER that was suppressed. The question is, how can this differentiation of the biphasic response be explained? The fact that the photoinactivation of Ntsr1-cells resulted in decreased single-unit responses in VPM during non-whisking and whisking in the absence of WER but had no effect on the early response component in VPM if conducted *during* WER, suggests that the S1L6 Ntrs1-projection to VPM experiences a physiological override under these conditions. One possibility is that the excitation of VPM, mediated by whisker deflection, overcomes the suppression effect (that was seen in the absence of whisking). This effect would be supported by sensory transmission through the leminiscal pathway, but also by the activation of the motor cortex, the deep layers of which excite S1L6 during active whisking.^59^ This finding also suggests that the S1L6 Ntsr1-pathway to VPM does not play an important role in response tuning to whisker deflection *onset* and thus, to deviant detection. Furthermore, this input is only relevant when whisker deflection occurs during active whisking. The effect of the photoinactivation of S1L6 Ntsr1-cells on the late component of the VPM response suggests, however, that this S1L6-VPM pathway may be especially important in the optimization of spatiotemporal item detection during an ongoing exploration event, especially given that the VPM expresses N-methyl-D-aspartate receptors and exhibits response facilitation during repeated corticothalamic activation.^60^ This interpretation aligns with the findings and interpretations of others.^61^^,^^62^

But what is the origin of this second component of the VPM response? Although the leminiscal pathway reaches VPM via the trigeminal nucleus that receives corticofugal projections from S1L2,^63^ this pathway is not likely to have contributed to the effects we report here, given that S1L6, and no other cortical layer, was affected by the photoinactivation of Ntsr1 cells under the conditions implemented. It has been argued that the early component of the VPM WER is regulated by S1L6 projections to the GABAergic reticular thalamic nucleus (TRN),^43^ which inhibits thalamic relay cells (such as the VPM).^64^^,^^65^ Li & Ebner^43^ reported that a local inhibition of S1L6, in urethane-anesthetized rats using muscimol, causes a suppression of the early component followed by an enhancement of the later component of WER in VPM.^43^ Here, however, the authors did not differentiate between cell types in S1L6, and one cannot exclude that urethane anesthesia altered cell responses.^66^ We think it is more likely that, in the case of the S1L6 Ntsr1 projection to VPM, it is the *late* component of the response that is affected by the TRN. For one, S1L6 Ntsr1 cells project directly to this structure,^36^^,^^67^ as do L5b neurons of the prefrontal cortex (PFC)^68^ and S1L5.^69^ The layer 5 corticofugal inputs to TRN have been ascribed a “driver” function^70^ given that they form very large synapses on parvalbumin (PV) positive-cells of TRN,^71^ meaning they can instigate very large depolarizations.^72^ The PFC is involved in feature-based attention, including toward somatosensory stimuli.^73^^,^^74^ Furthermore, it modulates activity in the sensory thalamus via TRN,^75^ and paired stimulation of the medial PFC and whiskers suppresses VPM WER.^76^ In addition, TRN and VPM have reciprocal connections.^77^^,^^78^ In principle, a reduction of Ntsr1 cell activity in S1L6 would result in reduced input to the TRN during WER, resulting in decreased activity of GABAergic neurons and thus decreasing inhibition of VPM. The outcome should be an *increased* second component of the VPM WER. However, the very strong single unit responses that we detected in VPM during whisking and WER suggest that both the PFC, S1L5, and the VPM inputs to the TRN will compete with the S1L6 Ntsr1 input and, under these conditions, *increase* excitation of the GABAergic TRN and thus suppress the second component of the VPM WER. TRN sends PV-cell projections to VPM.^79^^,^^80^ It has been proposed that the VPM input to TRN causes synchronized depolarizations of large populations of its cells,^81^ but can also suppress TRN activity related to whether preferred or adjacent whiskers have been deflected.^82^ Presumably, if the S1L6 Ntsr1-projection to TRN was intact, the outcome of this competition would be a boosting of attention to specific whisker deflections during whisking, given that VPM relays information about single whisker deflections to S1.^11^ This interpretation also suggests that under physiological circumstances, the S1L6 Ntsr1-projection to VPM subserves the fine-tuning and optimization of spatiotemporal aspects of whisker deflections.

### Photoinactivation of S1 layer 6 neurotensin receptor 1-expressing cells reveals differences in S1 layer 6 and medial posterior responses during whisker deflection

We have proposed above that the S1L6 Drd1 cell projection to POm supports deviant detection of whisker deflection, both during non-whisking (unexpected) and whisking (active seeking) phases, as well as the perception of subtle externally generated whisker movements. By contrast, we proposed that the S1L6 Ntsr1 cell projection to VPM supports spatiotemporal item detection during ongoing exploration (whisking) events. But S1L6 Ntsr1 cells project to both POm and VPM, raising the question as to the role of the S1L6 Ntsr1-POm input. As is the case for the S1L6 Drd1-POm input, photoinactivation of the Ntsr1-cell input suppressed single unit responses in POm to whisker deflection, during whisking and non-whisking phases. S1L6 Drd1-cell photoinactivation elicited a more potent suppression of WER during whisking compared to Ntsr1 cell inactivation. Interestingly, an interconnectivity between VPM and POm has not been identified thus far,^83^ indicating that activity in one of these nuclei does not directly impact the other. This suggests that the S1L6 Drd1-POm input may be the primary regulator of whisker deflection detection during active exploration. Nonetheless, POm is also influenced by the S1L6 Ntsr1 input, suggesting that this input may support the sensitivity of POm to whisker deflections, thereby increasing gain control, perhaps in support of the detection of subtle or weak whisker deflections. This might be the corticothalamic pathway through which POm supports the optimization of signal-to-noise ratios for stimuli that are unexpected, or perceived during phases of heightened attention, as well as stimulus predictions during active exploration, in line with functions for POm that were proposed by others.^16^^,^^22^

**Figure 4:**
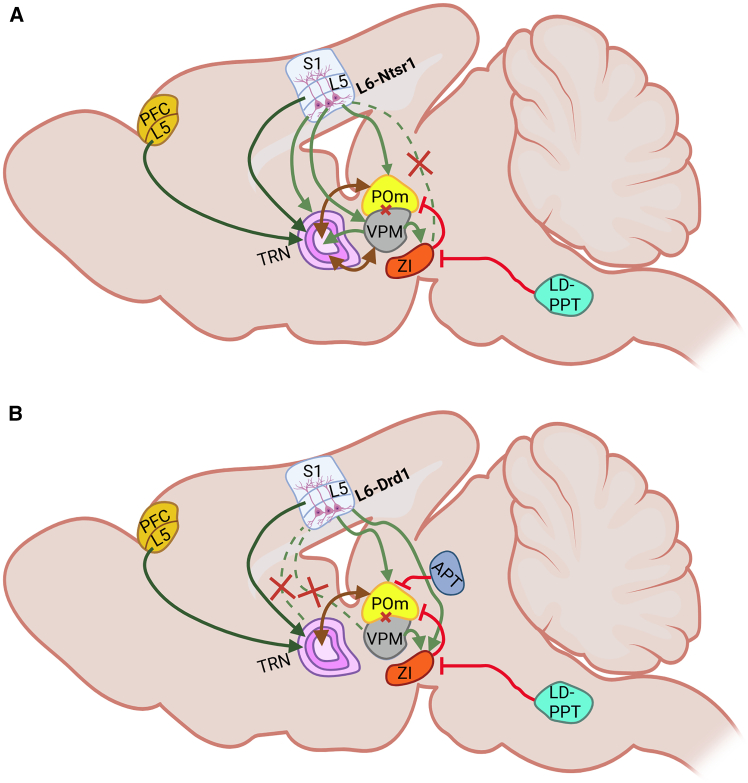
Putative regulation of distinct corticothalamic pathways originating in S1L6 The schemata offer two, as yet, unvalidated concepts as to how S1L6 Ntsr1-expressing or S1L6 Drd1-expressing cell projections to somatosensory thalamic relay neurons could be regulated. (A) S1L6 Ntsr1-expressing cells project to VPM, POm, as well as to the GABAergic thalamic reticular nucleus (TRN).^36^^,^^67^ VPM targets the middle layer of TRN but also sends a lower number of afferents to its core.^81^ TRN inhibits VPM, but VPM can either activate or inhibit TRN neurons, to prioritize information processing of preferred (VPM-TRN inhibition) versus adjacent whiskers (VPM-TRN excitation).^82^ Layer 5b of the prefrontal cortex and S1 also send corticofugal projections to TRN^68^^,^^69^ where they form “driver” synapses comprising large synaptic boutons, indicating that these inputs are capable of very potent depolarization of TRN neurons. Activation of PFC-L5 alters responses in both first order and higher order thalamic relay neurons.^75^ We propose that the excitation of VPM and TRN by S1L6 Ntsr1-expressing cells optimizes single (preferred) whisker perception, creating inhibition/excitation competition in TRN that is amplified by corticofugal inputs from layer 5. This subserves attention-based feature detection related to spatiotemporal somatosensory experience. The S1L6 Ntsr1-projection to POm subserves the optimization of signal-to-noise ratios,^83^ that is supported by a VPM input to zona incerta (ZI)^55^ and integration of inputs from other somatosensory thalamic relay populations in the reciprocal TRN-POm projection sites in the core of TRN.^81^ (B) S1L6 -expressing cells project exclusively to POm and do not project to TRN.^32^^,^^34^ Our data show that these cells project to ZI, which contains GABAergic neurons.^58^ Activation of ZI suppresses activity in POm.^54^ ZI is inhibited by neurons of the cholinergic laterodorsal tegmentum (LDT) and the pedunculopontine tegmentum (PPT) that are activated by increased arousal.^54^ POm is also subjected to inhibitory control by the anterior pretectal nucleus (APT),^84^ but its involvement in S1L6 Drd1-POm regulation is as yet unknown. We propose that the excitation of POm by the S1L6-Drd1 projection competes with the inhibition of POm, derived from the S1L6-Drd1 projection to ZI and the VPM-ZI projection. When ZI is activated, then the fine-tuning of deviant detection is modulated. With increased arousal (possibly triggered by the initial deviant detection), LD-PPT inhibits the ZI, allowing the S1 6 Drd1-POm pathway to prioritize stimulus predictions. Created using Biorender (https://BioRender.com/x720b0c).

Considering our current and previous findings, as well as those of others, we propose that a network including PFC and TRN, as well as S1L6 Ntsr1-cell projections to TRN and VPM, may play a significant role in the ability of VPM to engage in spatiotemporal somatosensory information processing (Figure 4). The S1L6 Ntsr1-pathway to POm may support its ability to detect weakly suprathreshold stimuli and modulate signal-to-noise ratios. We identified a novel projection pathway from S1L6 Drd1-cells to the GABAergic ZI and propose that S1L6 Drd1-cells regulate POm activity both through their direct corticothalamic projection, as well as through regulating the activity of ZI (Figure 4). This putative network could support the POm in detecting deviant as well as weak stimuli and may support the role for this structure proposed by others, in stimulus predictions and the integration of motor and somatosensory information. This proposal remains to be validated, and the role of the ZI in a somatosensory corticothalamic network needs to be clarified by further investigations.

### Conclusions

In this study, we describe the physiological responses of the cortex and thalamic relay neurons to two separate corticothalamic projections from S1L6 and identify a novel projection from S1L6 to ZI. Whereas Ntrsr1-expressing cells of S1L6 project to POm and VPM, S1L6 Drd1-cells project only to POm. Examination of single unit response during different somatosensory behavioral states revealed that the S1L6 Ntsr1-VPM pathway is especially tuned toward detecting whisker deflections during whisking states. The S1L6 Ntsr1-POm pathway heightens its sensitivity to whisker deflection, regardless of the whisking states, but the S1L6 Drd1-POm pathway supports a specialized sensitivity of POm to unexpected whisker deflections. Taken together, these findings suggest that corticothalamic control of the somatosensory thalamus is regulated, in a context-dependent manner, by two distinct S1L6 feedback systems that support the precision and fine-tuning of somatosensory perception.

### Limitations of the study

The whisker deflections that we implemented did not target preferred whiskers, which raises the question as to whether effects would be more sensitive if single whisker responses were monitored. It has been reported by others that single whiskers display frequency preferences that are reflected in signal responses of the barrel cortex.^85^ Thus, following an approach such as this could help better define the proposed role of VPM in detecting single whisker responses as mentioned above (Figure 4). Moreover, the origin of the biphasic VPM response to whisker deflection is, as yet, unknown, thus leaving inferred interpretations of the underlying circuitry open to debate. The precise role of the ZI in regulating POm function has yet to be elucidated. Here, too, the VPM input to the ZI, described by others, must be verified, due to the reported sparsity of this input.^53^ Finally, the identification of L6-Drd1 and L6-Ntsr1 cells in S1L6 was determined, on the one hand, by their recorded depth in S1L6, and on the other hand, on Arch-based-opto-tagging (see STAR Methods for details). This method is based on the detection of spontaneous firing rates, and therefore, L6-Drd1 and L6-Ntsr1 cells that exhibited very low spontaneous firing rates may have been classified as non-L6-Drd1 or non-L6-Ntsr1 cells. Thus, our findings may have excluded the role of low-spiking S1L6 neurons in corticothalamic information processing.

## Resource availability

### Lead contact

Further information and requests for resources and reagents should be directed to and will be fulfilled by the lead contact, Denise Manahan-Vaughan (denise.manahan-vaughan@rub.de).

### Materials availability

This study did not generate new unique reagents.

### Data and code availability

• Data: Pre-processed data used to make figures available in the main text or the supplemental information can be found at: https://doi.org/10.5281/zenodo.17455368.
• Code: This article does not report any original code.
• Other: Additional raw data is available from the lead contact upon request.

## Acknowledgments

We thank Ann-Christin Ammann and Hanna Lodwig for technical assistance and animal care, and Calvin Wu for providing additional software for the bwtt whisker tracking tool. This work was supported by a German Research Foundation (Deutsche Forschungsgemeinschaft, 10.13039/501100001659DFG) grant to D.M.V. (SPP 2411 - project number: 520284247). Pilot studies were funded by 10.13039/501100001659DFG grants SFB 874/A9 & B1 - project number: 122679504 to Patrik Krieger^†^ and D.M.V. The graphical abstract was created using Biorender (https://BioRender.com/9kat5ym). The authors declare no conflict of interest.

## Author contributions

Conceptualization: D.M.V. and J.A; data curation, formal analysis, methodology, and investigation: J.A.; validation, visualization, and data interpretation: D.M.V. and J.A.; funding acquisition: D.M.V.; project administration and supervision, resources: D.M.V.; writing-original draft and writing-review and editing: D.M.V. and J.A. Patrik Krieger († 2022) contributed to the original study concept, which was further developed by J.A. and D.M.V.

## Declaration of interests

The authors declare no competing interests.

## STAR★Methods

### Key resources table

**Table:** 

REAGENT or RESOURCE	SOURCE	IDENTIFIER
**Antibodies**		

Polyclonal rabbit anti mCherry	Invitrogen by Thermofisher	Cat# PA5-34974, RRID:AB_2552323
biotinylated-Goat-anti-rabbit	Vector Labs	Cat# BA-1000, RRID:AB_2313606

**Bacterial and virus strains**		

AAV-Flex-Arch-GFP, serotype 9	Addgene^86^	Cat# 22222-AAV, RRID:Addgene_22222
AAV2-EF1a-DIO-hChR2(H134R)-mCherry-WPRE-pA	UNC Vector Core	Dr. Karl Deisseroth, https://www.med.unc.edu/genetherapy/vectorcore/in-stock-aav-vectors/deisseroth/

**Chemicals, peptides, and recombinant proteins**		

Promega GoTaq® G2 Hot Start Master Mix	Promega	Cat# M7422
Kwik-Cast	World Precision Instruments,	adhesives/8633-kwik-cast
Bupivan (bupivacaine)	PUREN Pharma GmbH & Co. KG	PNZ 12675915
hydrogen peroxide	Sigma-Aldrich	Cat# H1009
Paladur	Kulzer GmbH	produkte/paladur
DiI	Life Technologies GmbH	Cat# D282
3,3’-diaminobenzidine with 1% nickel-ammonium-sulfate	BIOZOL Diagnostica Vertrieb GmbH	Cat# VEC-SK-4100

**Deposited data**		

Raw and analyzed data	This paper	https://doi.org/10.5281/zenodo.17455368

**Experimental models: Organisms/strains**		

Mouse Tg(Drd1-cre)FK164Gsat	Mutant Mouse Resource and Research Center (MMRRC)	Cat# 030781-UCD, RRID:MMRRC_030781-UCD
Mouse Tg(Ntsr1-cre)GN220Gsat	Mutant Mouse Resource and Research Center (MMRRC)	Cat# 017266-UCD, RRID:MMRRC_017266-UCD

**Oligonucleotides**		

Primer: Drd1a (30781) F1 (5’-3’) GCTATGGAGATGCTCCTGATGGAA	Invitrogen by Thermofisher	Custom DNA Oligonucleotide Synthesis Services
Primer: CreGS R1 (5’-3’) CGGCAAACGGACAGAAGCATT	Invitrogen by Thermofisher	Custom DNA Oligonucleotide Synthesis Services
Primer WT (5’-3’) CTCCAGAGCACATACTGACTCC	Invitrogen by Thermofisher	Custom DNA Oligonucleotide Synthesis Services
Primer: Ntsr1 F1 (5’-3’) GACGGCACGCCCCCCTTA	Invitrogen by Thermofisher	Custom DNA Oligonucleotide Synthesis Services

**Software and algorithms**		

BIOTACT (bwtt) whisker tracking tool	EU Framework 7 Project BIOTACT 215910^87^	https://sourceforge.net/projects/bwtt/
GraphPad Prism 9	GraphPad	https://www.graphpad.com
PAST 4.03	Hammer, 2001	https://past.en.lo4d.com/download
Clampfit	pClamp 9.2; Molecular Devices, LLC.	https://support.moleculardevices.com/s/article/Axon-pCLAMP-9-Electrophysiology-Data-Acquisition-Analysis-Software-Download-Page
Plexon Offline Sorter v3.3.5	Plexon Inc	https://plexon.com/products/offline-sorter/
Neuroexplorer	Nex Technologies	https://www.neuroexplorer.com/
ImageJ	Wayne Rasband (NIH)	https://imagej.net/ij/
ZEN 2	Carl Zeiss Microscopy	N/A

**Other**		

PCR Protocol Drd1	Mutant Mouse Resource and Research Center (MMRRC)	https://mmrrc.ucdavis.edu/protocols/030781Geno_Protocol.pdf
PCR Protocol Ntsr1	Mutant Mouse Resource and Research Center (MMRRC)	https://mmrrc.ucdavis.edu/protocols/017266Geno_Protocol.pdf
Franklin & Paxinos: Mouse brain atlas	Academic Press	ISBN 978-0-12-369460-7

### Experimental model and study participant details

All experiments were approved in advance by the ethics and animal welfare committee of the federal state of Northrhine-Westphalia (NRW) (Landesamt für Arbeitsschutz, Naturschutz, Umweltschutz und Verbraucherschutz, NRW) and followed the ARRIVE guidelines.^88^ The health status of the animals was monitored daily by the experimenter and/or animal care assistant and supported by weekly health checks by the veterinarian of Ruhr University Bochum. Humane end-points were implemented.

#### Animals

Animals were housed in a temperature- and humidity-controlled vivarium (Scantainer, Scanbur Technology A/S, Karlslunde, Denmark) that offered a constant 12-hour light-dark cycle (lights on: 8 a.m., lights off: 8 p.m.), *ad libitum* food and continuous water access. The two transgenic mouse strains used in this study were obtained from the Mutant Mouse Resource and Research Center (MMRRC) at University of California at Davis, an NIH-funded strain repository. These comprised Ntsr1-cre mice (Tg(Ntsr1-cre)GN220Gsat, RRID: 017266-UCD) and Drd1-cre mice (Tg(Drd1-cre)FK164Gsat/Mmucd, RRID: 030781-UCD). The Drd1-cre strain was donated to the MMRRC by Nathaniel Heintz, Ph.D., The Rockefeller University, GENSAT and Charles Gerfen, Ph.D., National Institutes of Health, National Institute of Mental Health.

The animals were bred as heterozygotes within the animal housing unit of Ruhr University Bochum (RUB). Within 3 weeks of birth genotyping was conducted by means of a small sample of ear tissue that was subjected to PCR. For Drd1-cre^+/+^ verification the following kit was used: Promega GoTaq® G2 Hot Start Master Mix (M7422), MMRRC's Strain Specific PCR protocol for 030781-UCD (UC Davis, Davis, CA, USA) with Primers for “Drd1”, “Cre” and “Wildtype”. For Ntsr1-cre^+/+^ verification the following kit was used: Promega GoTaq® G2 Hot Start Master Mix (M7422), MMRRC's Strain Specific PCR protocol for 017266-UCD (UC Davis, Davis, CA, USA) with Primers for “Ntsr1”, “Cre” and “Wildtype”. Male and female mice were used in the experiments. After treatment with adeno-associated virus (AAV), mice were housed single to prevent manipulations at the wound. Mice were handled daily, starting at postnatal day 14. Total individual animals used: 38 (18 Drd1-cre mice (8 males, 10 females) and 20 Ntsr1-cre mice (8 males, 12 females).

### Method details

#### Treatment with adeno-associated virus for optogenetics

Stereotaxically guided adeno-associated virus (AAV) injections were performed in 10, 4-6-month-old, Drd1-cre mice (4 males, 6 females) and 12 Ntsr1-cre mice (5 males; 7 females).

For this, Archaerhodopsin-3 (AAV-Flex-Arch-GFP, serotype 9, Addgene catalog nr 22222-AAV) was used.

Animals were first anesthetized with a ketamine (97 mg/kg) and xylazine (16 mg/kg) mixture, and their body temperature was kept constant at 37 °C using a heating pad (5 X 12.5 cm, 40-90-2-07, FHC Neural micro Targeting Worldwide, Bowdoin, ME, USA) connected to a temperature controller (DC Temperature Controller 40-90-8D, FHC Neural micro Targeting Worldwide, Bowdoin, ME, USA). Analgesia (4-5 mg/kg of Carprofen) was administrated subcutaneously. Animals were then placed in a stereotaxic frame (Model 1900, David Kopf Instruments, Tujunga, CA, USA). An incision in the skin over the skull midline was gently made, and the skull was exposed in the area where the injection was to be conducted. For injection, a small craniotomy (Ø 0.3 mm) was made, and the injection pipette lowered into the brain. Then, using a titer of at least 1×10^13^ vg/ml, 1 μl of Archaerhodopsin-3 was injected using the following coordinates, based on a mouse brain atlas^38^: anterior-posterior (AP) -1.7 mm from bregma and 3.1 mm lateral to the midline. The injection was made at a depth of 0.95 mm from the dura. After the injection, the craniotomy was covered with Kwik-Cast (World Precision Instruments, Friedberg, Germany) and the skin closed.

#### Headplate fixation

One week after AAV treatment, mice were anaesthetized as before with a ketamine/ xylazine mixture, for the purpose of attaching a head-plate to conduct awake head-fixed recordings. After anaesthesia had taken effect, the mouse was placed in a stereotaxic frame (SR-6M-HT, Narishige, Tokyo, Japan) and the analgesic ‘Marcain’ (Bupivan, bupivacaine (0.25 % (2.5 mg/ml), PUREN Pharma GmbH & Co. KG, Munich, Germany) was injected subcutaneously under the skin of the head. The previous incision was re-opened by removing the surgical stitches, and the wound and skull were cleaned with hydrogen peroxide (3 % in H_2_O; Sigma-Aldrich (H1009), Taufkirchen, Germany). The top of the skull was slightly roughened with a scalpel, and a custom-built head-plate was attached, above the intersection of bregma and lambda, using dental acrylic (Paladur; Kulzer GmbH, Hanau, Germany). The area above the somatosensory barrel cortex, VPM, and POm was not covered in dental acrylic, but instead covered with a low-viscosity silicone sealant (Kwik-Cast, World Precision Instruments, Friedberg, Germany), to allow easy access to the craniotomy. Animals rested in their home-cages for at least 10 days after this procedure.

#### Animal habituation and training

Animals were handled gently by the experimenter and/or animal care assistant prior to surgery, during daily health checks to begin habituation. Beginning after the recovery period from head-plate attachment, they were familiarised daily with head-fixation beginning with a 5 min period and increasing gradually to a 60 min fixation duration over a period of ca. 2 weeks. During fixation time, water was provided. Once the animal was comfortable with a 60-minute fixation duration, experiments were begun.

#### *In vivo* electrophysiology in awake mice

Recordings were conducted from the barrel cortex (S1 (L2/3-L6), POm and VPM) of awake mice. The day before the electrophysiological recordings were performed in awake mice, animals were anaesthetized with the ketamine / xylazine (16 mg/kg) mixture following the abovementioned procedure. The silicone sealant was removed and craniotomies (Ø 1 mm) for the electrophysiological recordings were made above VPM (AP: -1.8 mm, -1.5 mm lateral to midline) or POm (AP: -1.8 mm, -1.25 mm lateral to midline), and the craniotomy from the virus injection above somatosensory barrel cortex was enlarged (Ø 1 mm). A few drops of sterile physiological saline (0.9 % NaCl) were applied to the dura and the region was once more covered with silicone sealant. The next day, mice were head-fixed and extracellular recordings were obtained with 32-channel silicone laminar probes (NeuroNexus Technologies, Ann Arbor, MI, USA). For S1 these comprised: NeuroNexus laminar probe model A1x32-6mm-50-177, and for POm and VPM the laminar probe model A2x16-10mm-50-500-177 was used. The exact laminar probe positions were determined after the experiments, as described below. Data were acquired using a multichannel acquisition processor (Plexon Inc., Dallas, TX, USA). Electrophysiological recordings were conducted simultaneously in both S1 and VPM and POm using two separate multi-channel electrodes (Figure 1). The silicon probes used for recording VPM, or POm responses were placed in an area where photoinactivation of L6-Drd1 or L6-Ntsr1 cells caused a short latency decrease in action potential spiking. Recordings from the S1 and VPM, or S1 and POm regions were thus aligned according to their corticothalamic responses.

The raw signal (acquired at 40 kHz) was bandpass-filtered between 300-3000 Hz and signals that exceeded the background noise by at least 4 standard deviations (SDs) were designated as action potential spikes. To build peristimulus time histograms (PSTHs), the spikes were summed over all recorded sweeps and divided into whisking, or non-whisking episodes. Spike sorting was conducted with ‘Offline Sorter’ software (Plexon Inc, Dallas, TX, USA), using the T-Dist E-M method (T-Distribution with Expectation Maximization algorithm; outlier Threshold 1.00; degrees of freedom: 2.00), as previously described.^32^ Units were considered single-units when a significance (p) level of < 0.05 was reached according to 3D principal component analysis (peak amplitude asymmetry, trough to peak latency and spike half-width). Single-units recorded in S1L6 that responded with a decreased averaged spontaneous activity in the first 5.5 ms following photoinactivation (determined from juxtacellular recordings with subsequent cell identification that was lower than the mean + 2 SD (calculated from 200 ms preceding commencement of photo-inhibition), were classified as units recorded from S1L6-Drd1, or S1L6-Ntsr1 cells (L6-Drd1: n = 46, L6-Ntsr1: n = 64). This strategy was based on previous validations conducted by former colleagues of our lab, using the same intrastructure and methods described here: They reported a latency 5.5 ± 0.77 ms (mean ± SEM) for single-unit response decreases, following commencement of photoinactivation.^37^

WER response latency was calculated as described before.^32^ For each individual cell, the spontaneous activity (under control conditions) that preceded whisker stimulation was averaged over 200 ms (2 ms bins). We then compared the activity *after* the whisker stimulation onset in each bin with the averaged spontaneous activity recorded prior to whisker stimulation for that specific cell. If the activity was higher than the spontaneous activity plus twice the SD (for a minimum of 5 subsequent bins), then we estimated the onset of the WER as comprising the first time point when this criterium was reached (latency VPM WER1 response: ∼5 ms, WER2 response: ∼41 ms; latency POm WER response: ∼6 ms; latency Drd1 WER response: ∼8 ms; latency Ntsr1 WER response: ∼6 ms). In POm, a prolonged WER could be detected in some cells, that exhibited no clear second peak and appeared between 50 ms to 150 ms after whisker stimulation onset. Since this effect was not consistently present in all recorded cells, the analysis of this prolonged response was not reported in the main part of this paper (see supplemental information for further details of this analysis).

Spike-sorted units were analysed by means of cross- and autocorrelations to ensure that they were separate units (Table S8) (NeuroExplorer V5; Nex Technologies, Colorado Springs, CO, USA). Fast-spiking putative interneurons were distinguished from excitatory cells in the cortical layers, based on 3D-principal component analysis (Units with each of the following criteria were considered as interneurons: peak amplitude asymmetry >0.15; Trough to peak latency < 0.3 ms; spike half-width < 0.2 ms^89^) and any interneurons that were detected were excluded from the analysis (Figure S7).

The experimental protocol was as follows. Thirty sweeps of stimulation with each of the following protocols: (i) photo-inhibition only, (ii) air-puffs only, applied as 25 deflections with 20 ms in duration, repeated at a frequency of 0.5 Hz, (iii) photo-inhibition in combination with air-puffs (applied, as in ii). The order was randomized within and between each experiment. The entire cycle was repeated at least twice and maximally thrice. During the offline analysis, the sweeps were (as described above) divided into “whisking” or “non-whisking” events. The number of recordings of the response to photo-inhibition only, to air-puffs only, and to the combination of both, for the S1L6-Drd1 data (10 experiments) was 25, 25 and 25 in the non-whisking condition, and 22, 25, 25 during whisking, respectively. For the S1L6-Ntsr1 data (12 experiments) the number of recordings of the response to photoinactivation only, to air-puffs only, and to the combination of both, was 24, 26 and 24 during non-whisking, and 20, 25, 26 during whisking, respectively. Every cell recorded from the same animal experienced the exact same number of recorded sweeps per condition. For each animal, only one experiment (recording session; ∼60 min) was performed. See Table S9 for recorded trials for each experiment and each cell.

Single-unit activity (SUA) was divided by the number of recorded sweeps from the same brain state. Thus, if for example, a total of 100 spikes were measured in thirty sweeps calculated in a one second time window in each sweep, the result would be 3.33 spikes/s (100 spikes/30 sweeps). The spontaneous or baseline spiking activity that preceded the whisker deflection was analysed from the beginning of photoinactivation until the commencement of whisker deflection. To differentiate spiking activity during whisking from that during non-whisking events, the former is referred to as “whisker-related spiking” activity and the latter is described as “random spontaneous” activity. Thus, in both cases spiking activity was recorded when the whiskers were not being deflected with air-puffs.

#### Whisker stimulation and tracking

To stimulate the whiskers contralaterally to the recording site, an air-puff device (PDES-O2DX, npi electronic instruments for the life sciences, Tamm, Germany) was triggered by an electronic stimulation interface (‘Master-8’, A.M.P.I., Jerusalem, Israel). Air-puffs of 20 ms duration and 1.12 bar intensity were applied to the proximal (to body) part of the whiskers at a frequency of 0.5 Hz. Mice were not trained beforehand to receive this kind of stimulation. Previous determination of the stimulus-response relationship in anesthetized mice,^32^ indicated that the 1.12 bar air-puff is at the bottom plateau for detection of a whisker-evoked increase in action potential spiking. The time from onset of the trigger pulse until the air flow reached the whiskers was measured with a piezo pressure sensor (EPZ-27MS44W Piezoelement, 4.4 kHz, 200 Ohm, Reichelt Elektronik, Sande, Germany) and estimated to comprise ca. 10 ms.

Air-puff deflections of multiple whiskers, (∼15 whiskers; estimated based on offline analysis of video recordings (Pike F-032B, ALLIED Vision Technologies GmbH)), rather than estimations of the deflection of a single whisker, were used here.

Air-puff deflection of whiskers was externally applied during different whisking phases. The action potential spiking activity was continuously recorded, and the data were divided into two categories: spiking activity occurring when the mouse was not using its whiskers (i.e., non- whisking episodes), or spiking activity occurring when the mouse was actively whisking. In all cortical layers, as well as in the thalamus, the spiking activity of excitatory cells increased during whisking compared to non-whisking episodes (Table S1).

Thus, whisker deflection during episodes classified as “whisking” or “non-whisking”, correlated with increased activity.

Whether the animal was either whisking or not whisking during the experimental protocol was determined offline, manually, on a frame-by-frame basis from videos recorded simultaneously with the electrophysiology recordings (208 fps), using the “BIOTACT (bwtt) whisker tracking tool” (Figure S8).^87^ The person doing this classification did not know which genotype was tested, or which stimulation protocol was used. The episodes thereby classified as either “whisking,” or “non-whisking”, were verified with a spectrogram analysis of the local field potential (LFP), following an approach established by Urbain and colleagues.^49^ For this, the raw LFP of each individual channel (and for each individual recording) was exported to Neuroexplorer (NeuroExplorer V5; Nex Technologies, Colorado Springs, CO, USA). Here, the spectrogram analysis function for continuous data was used to plot each individual spectrogram. The PSD colour (Power-Spectrum-Density) was plotted as log of PSD (db) and, for the FFT (Fast Fourier Transformation), the Welch function was chosen. The numerical results related to time were exported to excel and here, timestamps were sorted into episodes of whisking and non-whisking. Brain-state episodes were sorted as described before^49^:

“quiet” non-whisking state (4-12 Hz), “active” non-whisking and whisking state (13-30 Hz; 25-60 Hz). Episodes classified as “active non-whisking” were removed from analysis and not shown, since they were too short in time to represent a specific brain state during baseline and whisker-evoked activity recording. Therefore, this publication only reports the “quiet” non-whisking state (referred as non-whisking, and containing only sweeps where whisker stimulation does not promote self-induced whisking) and the “active” whisking state (referred to as whisking).^49^

#### Photoinactivation of transfected cell populations

To inactivate L6-Drd1-cre or the L6-Ntsr1-cre cells, via light stimulation of Archaerhodopsin-3, an LED module that generated light in the 550 nm wavelength (lime green) was used. The output power of the LED driver (PlexBright LD-1, Plexon Inc., Dallas, TX, USA) was set at an intensity of 4.53 mW (35.83 mW/mm^2^), corresponding to 250 mA, was regulated via the current adjust knob. The power output at the fibre tip was measured with a power meter (PM100D; Thorlabs, Newton, NJ, USA).

For optogenetic control of the S1L6-Drd1 and S1L6-Ntsr1 corticothalamic cells, an optical multimode fibre (400 μm, 0.50 numerical aperture (NA), model: M128L01, Thorlabs, NJ, USA) was placed on the surface of the vibrissal primary somatosensory barrel cortex (AP: -1.7 mm from bregma and 3.1 mm lateral to the midline). Once the electrodes were lowered to the correct depth in the somatosensory barrel cortex (tip just below the white matter), and POm or VPM (tip of silicon probe at depth 3.7 – 4.1 mm; as confirmed with postmortem histology), action potential spiking activity was recorded in control (no photoinactivation), or during photoinactivation to inactivate S1L6-Drd1 or S1L6-Ntsr1 cells (randomized protocol order, see section *in vivo* electrophysiology recordings). Spiking activity during photoinactivation only (no air-puffs) was calculated for the duration of the light pulse. As a control, the optogenetic inactivation strength between S1L6-Drd1 and S1L6-Ntsr1 cells were compared (Table S10) and showed similar levels of inactivation.

#### Verification of AAV transfection and electrode localization

After the *in vivo* electrophysiology experiments, animals were euthanized with ketamine (97 mg/kg) / xylazine (16 mg/kg) and transcardially perfused with phosphate-buffer followed by 4 % PFA (paraformaldehyde). After post-fixation for 48 h the brains were sliced coronally in 100 μm slices. Correct virus expression was confirmed with microscopy (Figure S9). Since the laminar probes were dipped into DiI before the start of the experiments, the trajectory of the probes could be confirmed using the DiI labelling (D282; Life Technologies GmbH, Germany; Figure 1) and a brain slice alignment with the Franklin & Paxinos atlas. The layer position of the electrodes / channels was then determined based on the probe trajectory and the layer borders. Layer borders were determined as described by others.^90^ The following borders were used: forS1 L2/3: 65-319 μm; for S1L4: 320-539 μm; for S1L5: 540-774 μm and for S1L6: >775 μm.^90^ In addition, the layer borders were confirmed by the characteristic shape of the local field potential.^91^ The projecting areas from S1L6-Drd1 and S1L6-Ntsr1 cells to POm were confirmed with histological analysis, as previous described.^32^

#### anti-mCherry-DAB-Ni immunohistochemistry

To investigate projection targets of the S1L6-corticothalamic cell populations, cortical injections of double-floxed-mCherry with subsequent anti-mCherry-DAB-Ni antibody-staining, was performed to visualize S1L6-terminals. For this, stereotaxically guided AAV injections were performed in eight, 4-6-month-old, Drd1-cre mice and eight Ntsr1-cre mice, as described above. Three weeks later, mice were euthanized with a ketamine (97 mg/kg) and xylazine (16 mg/kg) mixture and transcardially perfused with phosphate-buffer followed by 4 % PFA (paraformaldehyde). After post-fixation for 48 h, brains were transferred to cryo-protection-solution and ∼1 week later sliced coronally in 30 μm slices. Next an antibody-staining against mCherry (Thermo-Fisher Scientific Inc., Massachusetts, USA) with subsequent DAB-Ni-immunohistochemistry (3,3’-diaminobenzidine with 1% nickel-ammonium-sulfate (BIOZOL Diagnostica Vertrieb GmbH, Eching, Germany) was done. Stained brain-slices were interpreted according to a mouse-brain atlas,^38^ using an semi-automated algorithm in Fiji (ImageJ; Wayne Rasband (NIH)).

### Quantification and statistical analysis

All data are reported as mean ± SEM, unless stated otherwise. Data was tested for normal distribution using the two-sample Kolmogorov-Smirnov test (α = 0.05). Statistical analysis of changes in spiking activity was done using a two-way analysis of variance (ANOVA) with repeated measures (GraphPad Prism 9; GraphPad Software, Boston, MA, USA) considering the row factor as within factor of one group (e.g. “spont” vs. “WER”) and the column factor as between-factor condition different groups (e.g. “control” vs. “opto”) unless stated otherwise. Outliers were identified by means of ROUT with Q = 1% (GraphPad Prism 9; GraphPad Software, Boston, MA, USA) and excluded from analysis. Additional statistical analysis (PAST 4.03; Øyvind Hammer, Oslo, Norway^92^) of e.g. absolute changes in spiking activity were done by using one-sample *t*-tests (theoretical mean is zero). Subsequently Bonferroni corrected *p*-values (p values multiplied by the number of comparisons) were used to further correct for multiple testing. Analysis of the whisker-evoked response slope were done in Clampfit (pClamp 9.2; Molecular Devices, LLC., San Jose, CA, USA). To analyse the slope of the whisker-evoked response dynamics, the PSTH from each individual cell was plotted separately. The PSTHs were then identified according to a corresponding time column and the slope was analysed in spikes/s using the slope analysis function in Clampfit (pCLAMP9.2; Molecular Devices, LLC., San Jose, CA, USA). The slope was measured from the beginning of the evoked response until its maximum peak.
