# Supplementary material for: Somatosensory control of thalamic relay neurons is regulated by two distinct layer 6 feedback systems
Source: iScience. 2025 Dec 12;29(1):114427. doi: 10.1016/j.isci.2025.114427 (PMC12796580; doi:10.1016/j.isci.2025.114427)
Supplement: Document S1. Figures S1–S9, Tables S1–S10, and Data S1 and S2 [file mmc1.pdf]

iScience, Volume *29*

Supplemental information

Somatosensory control of thalamic relay neurons is regulated by two distinct layer 6 feedback systems

Josephine Ansorge and Denise Manahan-Vaughan

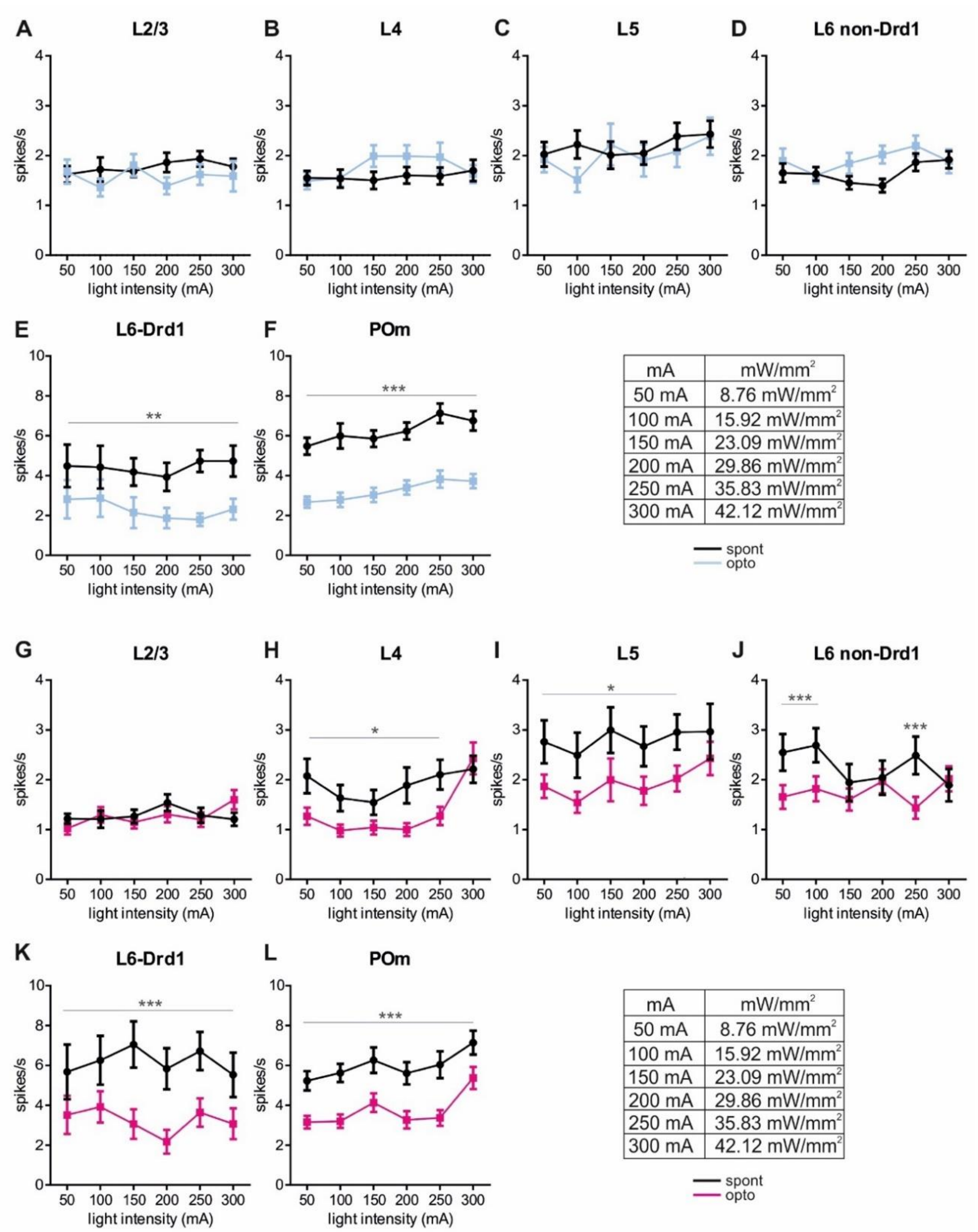

Fig. S1 Spiking activity during optogenetic inactivation of S1L6-Drd1-expressing cells using different light intensities and pulse durations

(**A-F**) Panels show the effect of photoinactivation of Drd1-expressing cells in S1L6 on spike responses recorded in cortical layers L2/3 (**A**), L4 (**B**), L5 (**C**) in L6 (**E**), as well as in POm (**F**). Here, a light pulse (550nm) of 70 ms duration was applied at different light intensities (50 - 300mA) to stimulate Archaerhodopsin that had been transfected 3 weeks previously into S1L6. The spikes are averaged over a 70 ms time window, corresponding to light application. “S1L6 non-Drd1” (**D**) shows responses in three cells that were identified as outliers and excluded from analysis (with ROUT, Q = 1%).

(**G-L**) Panels show the effect of S1L6-Drd1-cell photoinactivation on electrophysiological responses recorded in cortical layers, L2/3 (**G**), L4 (**H**), L5 (**I**) in L6 (**K**), as well as in POm (**L**), using a light pulse of 120 ms duration at different light intensities (50 - 300mA). The spikes are averaged over a 120 ms time window. In panel **J** (“L6 non-Drd1”) the same three cells were excluded as in panel **D**.

Data are reported as mean ± SEM from single units, asterisks represent significant differences between spontaneous (spont.) activity and responses recorded during photoinactivation (opto); one-way rmANOVA). *p<0.05, **p<0.01, ***p<0.001. (This figure provides extended data related to Figure 1 of the main manuscript).

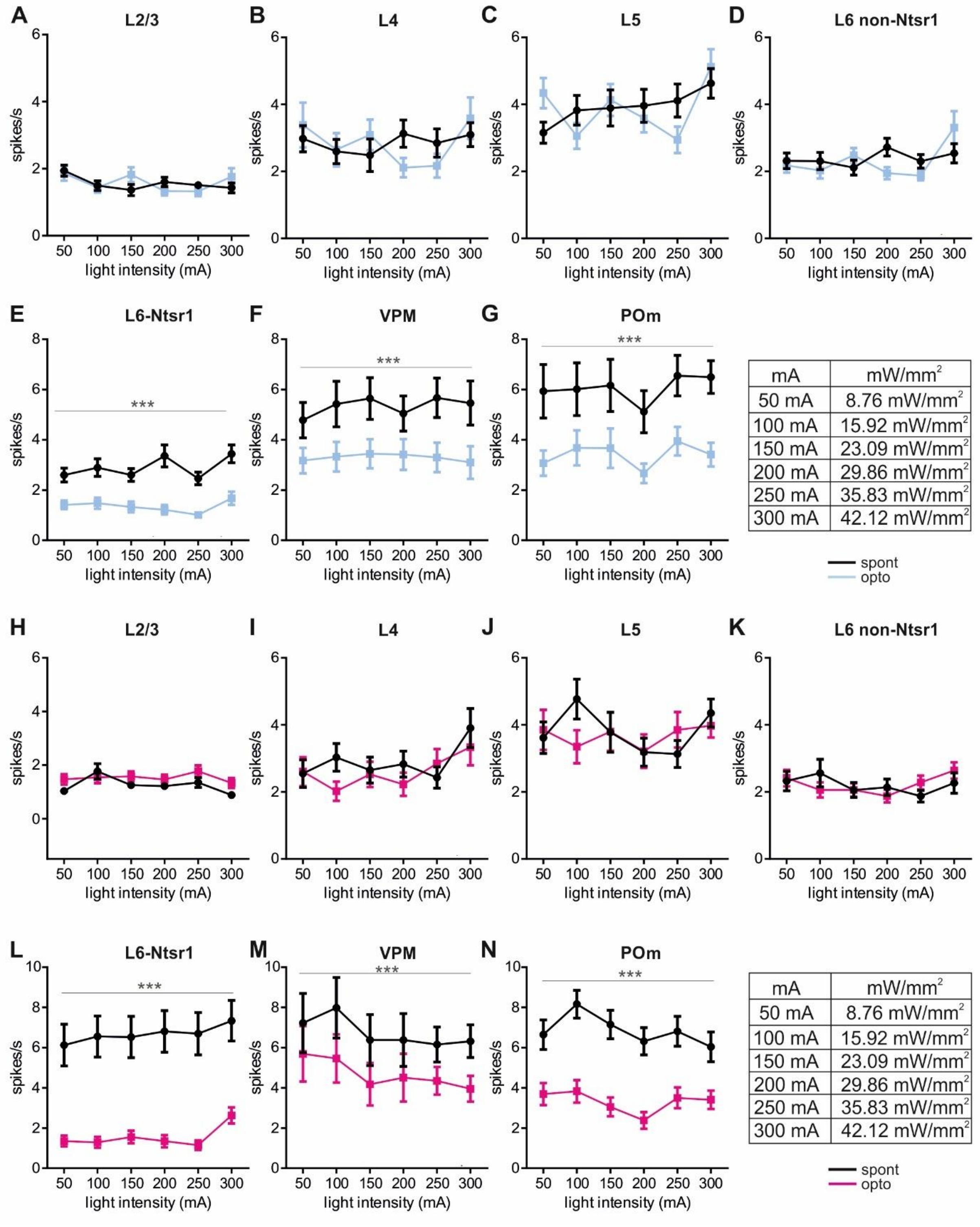

Fig. S2 Photoinactivation of S1L6-Ntsr1-expressing cells by stimulation of Archaerhodopsin using different light intensities and pulse durations
(**A-G**) Panels show the effect of photoinactivation of S1L6-Ntsr1-expressing cells on the other cortical layers, in VPM and POm using a 70 ms long pulse (550nm), at different light intensities (50 – 300mA). (**H-N**) Panels show the effect of photoinactivation of S1L6-Ntsr1-expressing cells, with a light pulse of 120 ms duration, on electrophysiological responses recorded in the other cortical layers, as well as in VPM and Pom (light intensities: 50 - 300mA). Data are reported as mean ± SEM from single units, asterisks represent significant differences between the spontaneous (spont.) activity and responses recorded during photoinactivation (opto); one-way rmANOVA). *p<0.05, **p<0.01, ***p<0.001. (This figure provides extended data related to Figure 2 of the main manuscript).

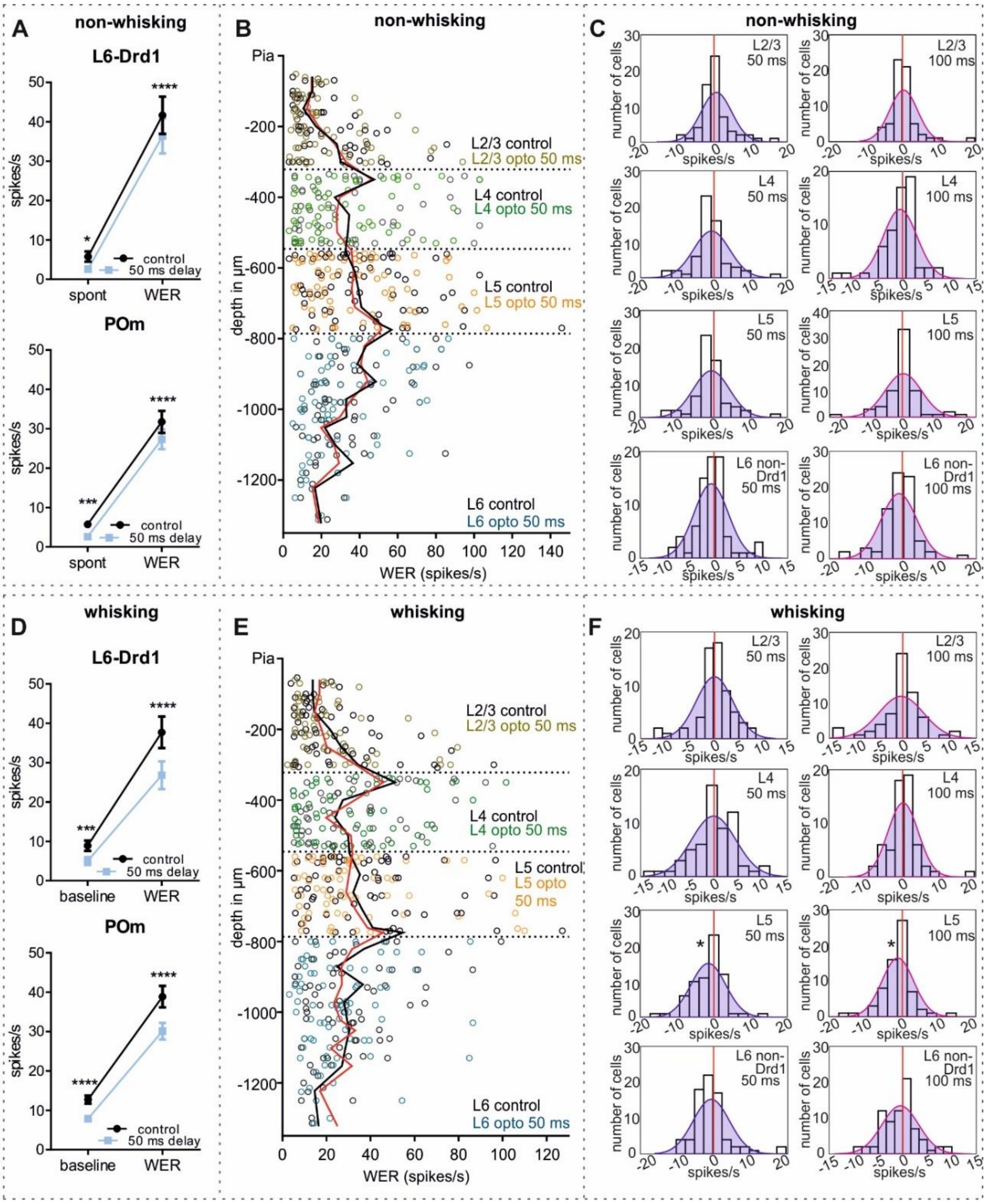

Fig. S3 Effect of photoinactivation of S1L6-Drd1-expressing cells on the whisker-evoked response (WER) in somatosensory barrel cortex and POm during non-whisking and whisking episodes

(**A**) Panels shows the spontaneous (spont) minus WER responses for S1L6 and POm cells (**B**) Panels show WER responses of each cell with (opto) and without photoinactivation (control); x-axes: WER, y-axes: recorded depth in barrel cortex, control = activity recorded without light application, and opto = activity recorded with S1L6-Drd1 cell inactivation. Black line signifies average WER in controls per layer, separated into in 50 µm bins. Red line signifies average WER per layer (in 50 µm bins) during photoinactivation of S1L6- Drd1 cells.

(**C**) Histograms of the "WERopto minus WER" (data from B) from the different cortical layers; x-axis: "WERopto minus WER" spikes/s).

(**D**) same plot as (**A**), but for the whisking condition.

(**E**) same plot as (**B**), but for the whisking condition.

(**F**) same plot as (**C**), but for the whisking condition (data describe mean ± SEM from single units). **C, F**: S1L5 shows a trend towards decreased activity when WER is applied during whisking compared to non-whisking phases, as reported by others [1], this reduction becomes significant (*) during photoinactivation of Drd1-expressing cells. (This figure provides extended data related to Figure 3 of the main manuscript).

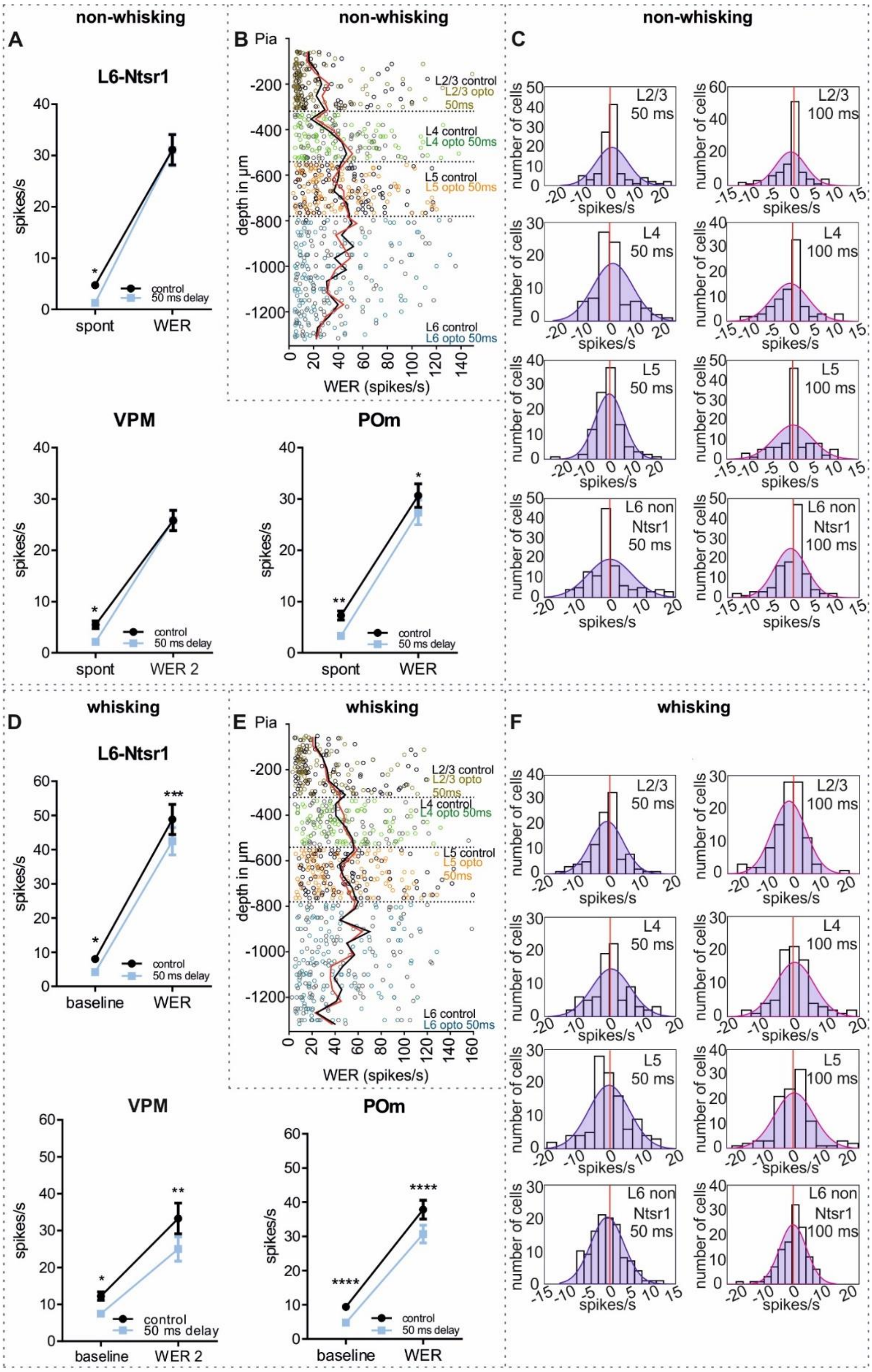

Fig. S4 Effect of photoinactivation of S1L6-Ntsr1-expressing cells on the whisker-evoked response (WER) in somatosensory barrel cortex, VPM and POm during non-whisking and whisking episodes

Panels show spontaneous activity (spont.)–WER responses for S1L6-Ntsr1 cells, VPM and POm in non-whisking (**A**) and whisking conditions (**D**). WER for each individual cell is shown in panels **B** (non-whisking) and **E** (whisking).

(**C, F**) Histograms of the "WERopto minus WER" (data from B and E) from the different cortical layers. (data are reported as mean ± SEM from single units). (This figure provides extended data related to Figure 3 of the main manuscript).

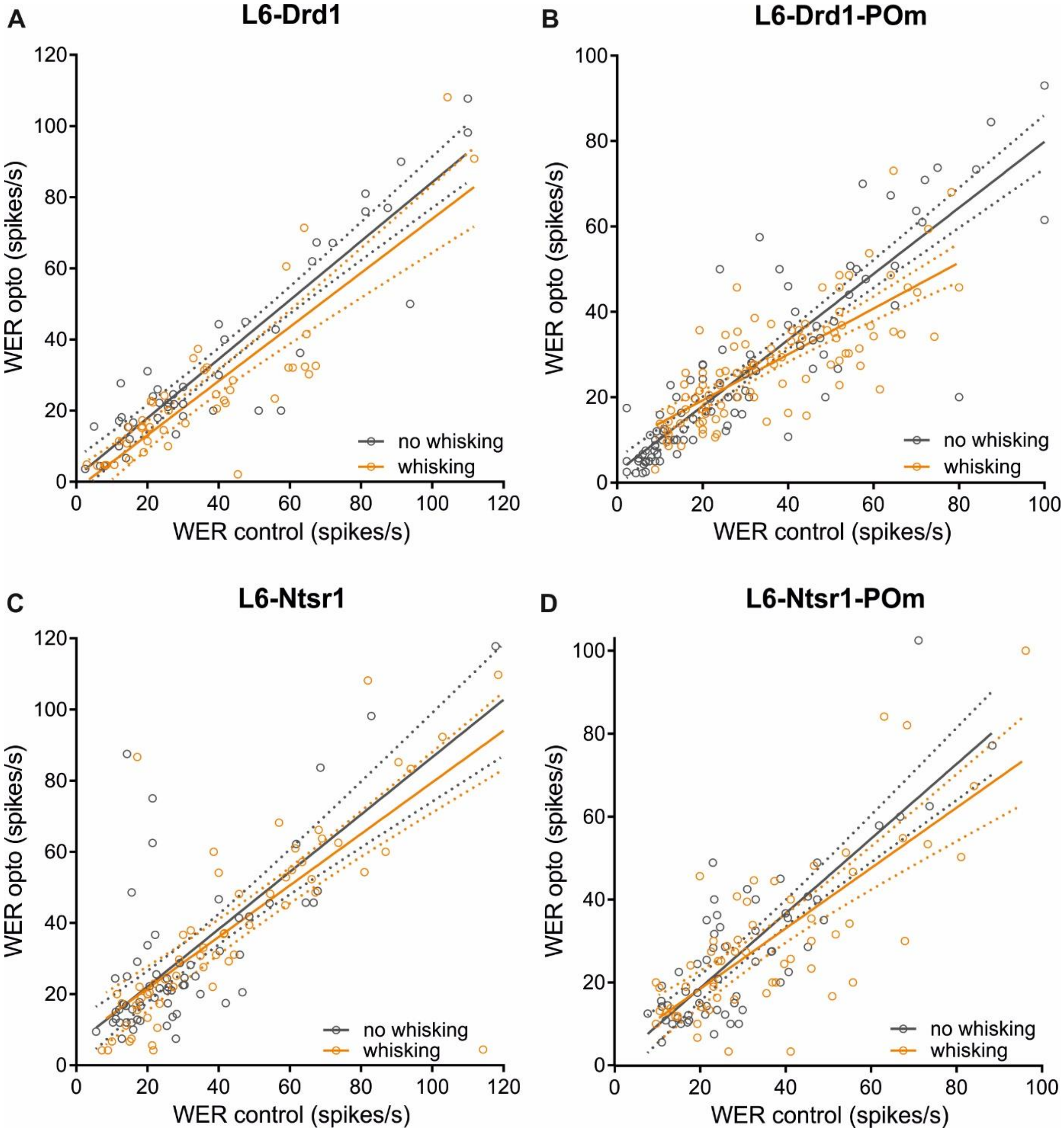

Fig. S5 Linear regression plots of the whisker-evoked response (WER) during non-whisking and whisking episodes

The panels show the individual linear regressions of WER to compare the slopes between non- whisking (grey line) and whisking episodes (orange line) (x-axis: WER in control; y-axis: WER during photoinactivation; mean ± 95% CI. Equations and R^2 values are reported in the first paragraph of the Supplementary Information (below). (**A**) Panel shows the linear regressions in S1L6-Drd1 cells and in connected POm cells (**B**). (**C**) The panel reports the linear regressions in L6-Ntsr1-cells and in connected POm cells (**D**). (This figure provides extended data related to Figure 3 of the main manuscript).

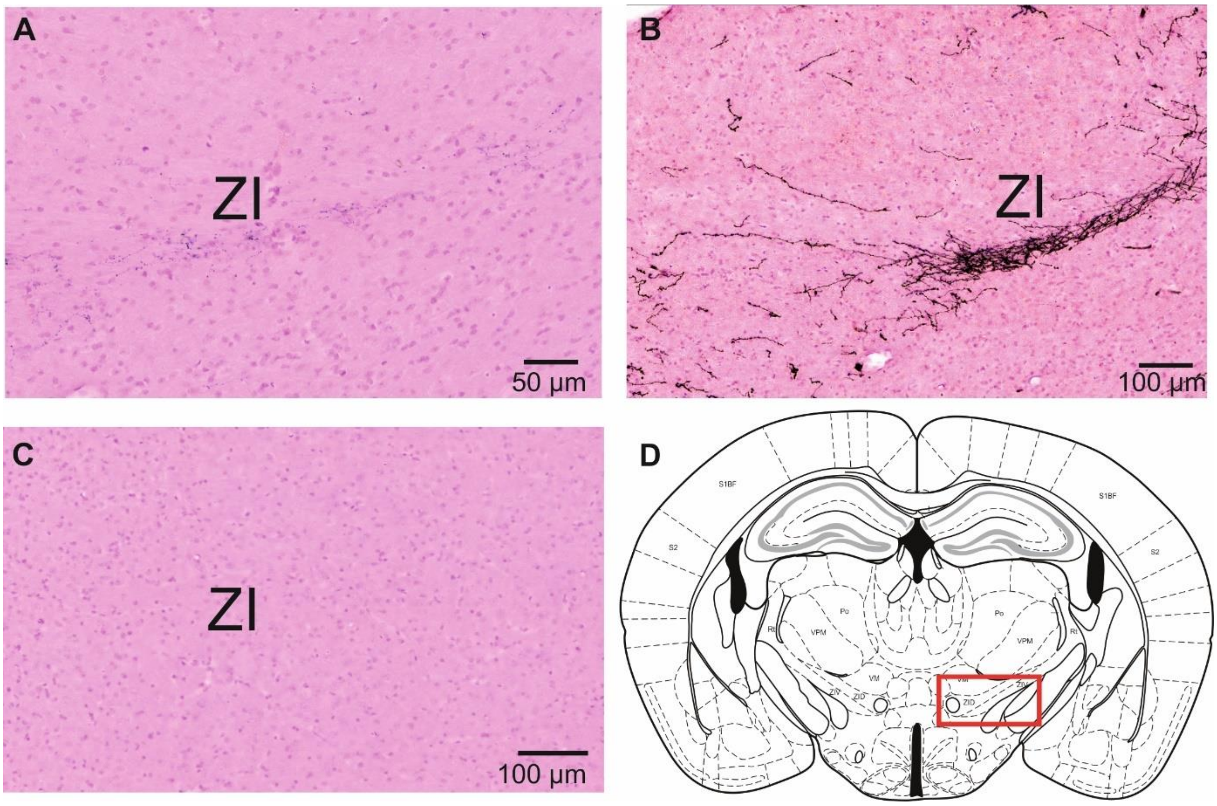

Fig. S6 Terminals of S1L6 inside zona incerta
Drd1-cre and Ntsr1-cre mice were injected with mCherry into S1L6. The mCherry signal was amplified using DAB-Ni with horseradish counterstaining.
(**A**) L6-Drd1 fibers detected inside the zona incerta of a Drd1-Cre mouse.
(**B**) L6-Ntsr1 fibers inside the zona incerta (ZI) of an Ntsr1-Cre mouse.
(**C**) Image of a negative control from one mouse. Here, the tissue was treated only with blocking medium (normal goat serum in PBS) without using the first antibody. The subsequent tissue treatment of the immunohistochemical preparation was the same as for A and B.
(**D**) Corresponding brain atlas image [2]; red rectangle shows region visualized in A-C. (This figure provides extended data related to Figure 3 of the main manuscript).

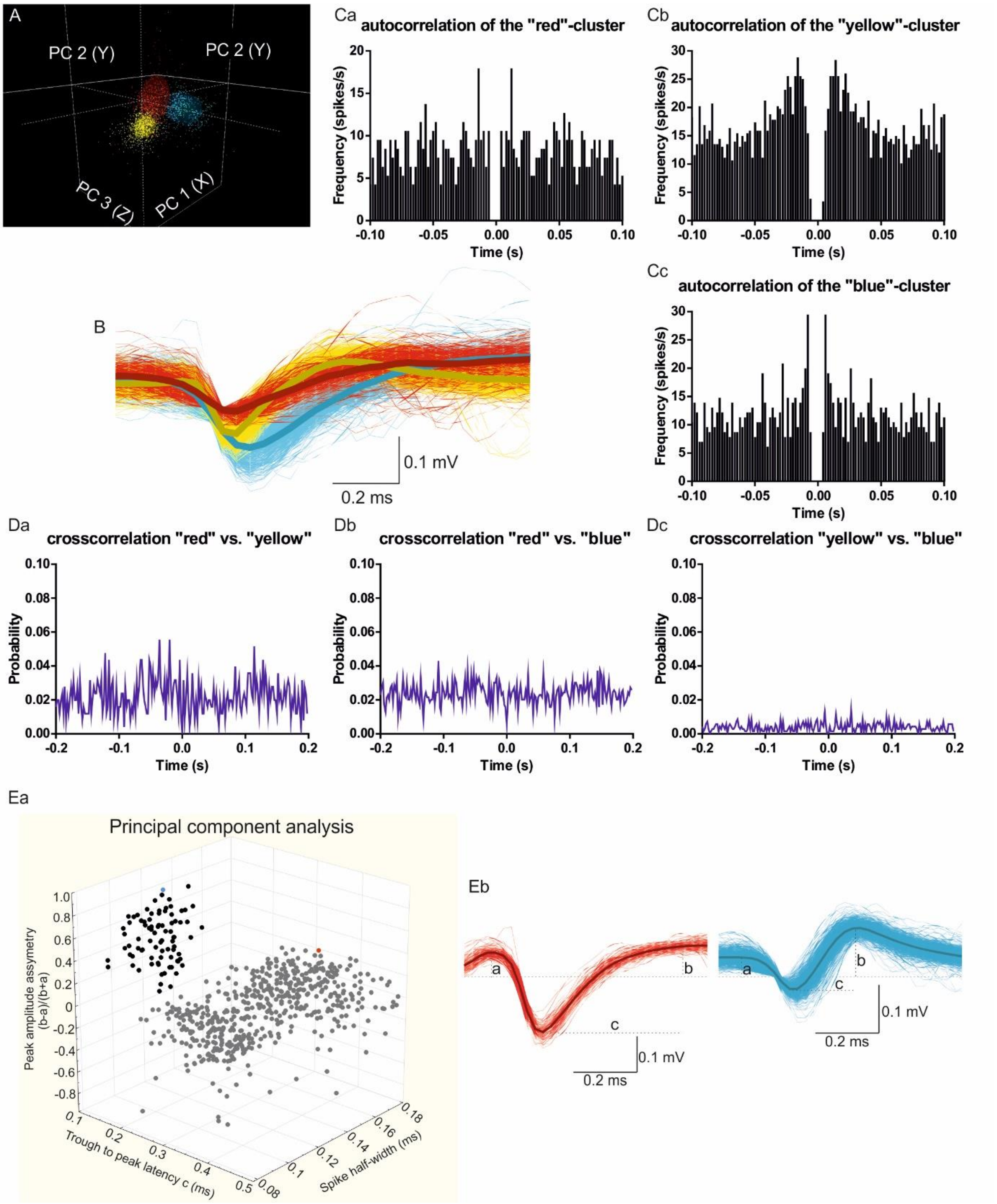

Fig. S7 Spike-sorting analysis

(**A**) 3D-Cluster of three individual recorded units represented in red, blue and yellow.

(**B**) Waveform of recorded units shown in (A).

(**Ci-Ciii**) Autocorrelelograms of the recorded units shown in (A).

(**Di-Diii**) Crosscorrelelograms of the recorded units from (A).

(**E**) 3D-Principle-Component analysis of all recorded units. The three components are: peak amplitude asymmetry, trough to peak latency, spikes half-width (ms).

(**F**) Example waveforms of two spike-sorted units. In red, a putative fast-spiking interneuron and, in blue, an excitatory neuron are shown.

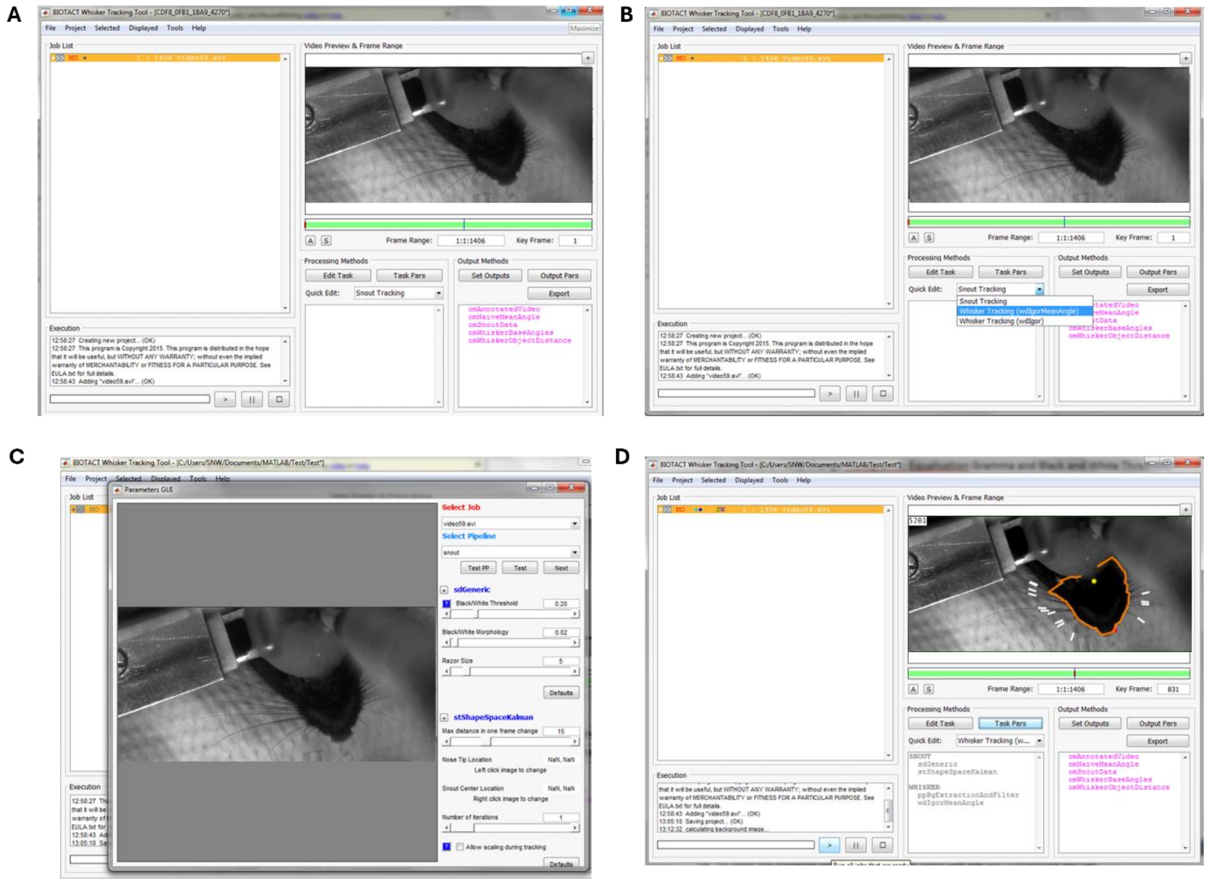

Fig. S8 Whisker tracking with the bwtt whisker tracking tool

(**A-D**) Images showing the workflow using the whisker tracking tool [3] to track the whisker angle. This is as follows:

(**A**) Choose a video file and load it (it will look like **A**).

(**B**) Select “Whisker Tracking (WdIgorMeanAngle)”.

(**C**) For “Select Pipeline” choose “snout” and (using the mouse) mark the snout tip and then the centre of the head.

(**D**) Start the whisker tracking process.

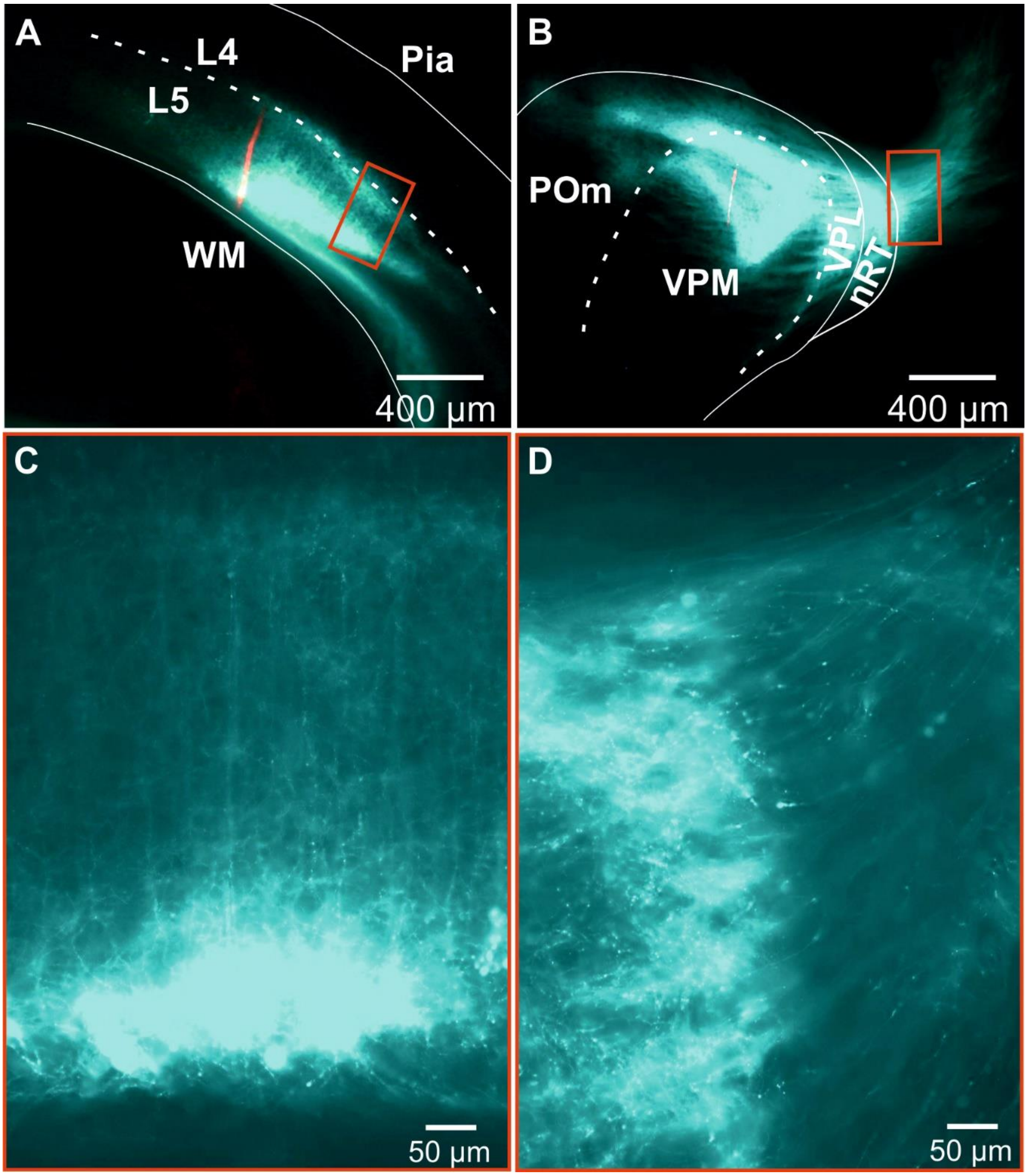

Fig. S9 Example of the Archaerhodopsin-GFP expression in layer 6 Ntsr1-expressing cells
(**A-B**) Archaerhodopsin-GFP expression in S1L6 of Ntsr1-cre mouse (same example as in **Figure 1 E, F** of main manuscript), as detected using immunohistochemistry. Red rectangles indicate region which was used to create the “zoom-in” images shown in (C) and (D).
(**C**) zoomed image of the S1 region shown in (A).
(**D**) zoomed image of the thalamic region shown in (B).

	L2/3	L4	L5	L6	L6-Drd1	L6-Ntsr1	POm	VPM
	Spikes/s no whisking							
Spont.	2.99 ± 0.44	3.64 ± 0.50	3.49 ± 0.44	1.67 ± 0.26	4.79 ± 0.87	4.08 ± 0.51	6.12 ± 0.44	5.47 ± 0.74
WER	22.53 ± 1.84	37.26 ± 2.19	42.95 ± 2.17	22.53 ± 1.84	41.68 ± 4.74	31.11 ± 48.83	31.35 ± 1.96	25.80 ± 1.99
	Spikes/s whisking							
Spont.	5.81 ± 0.66	5.89 ± 0.60	5.95 ± 0.51	5.60 ± 0.50	8.92 ± 1.32	8.02 ± 0.88	11.42 ± 0.74	12.25 ± 1.12
WER	31.73 ± 2.34	42.34 ± 2.46	48.85 ± 2.48	31.73 ± 2.34	37.70 ± 4.01	48.83 ± 4.42	38.38 ± 2.01	47.23 ± 4.04
Two-way ANOVA	p-value							
Non-whisking vs. whisking	**< 0.0001** F(1,163)=28.81	**0.0059** F(1,142)=7.815	**0.0082** F(1,170)=7.156	**0.0006** F(1,200)=12.09	**<0.0001** F(1,45)=71.58	**0.0073** F(1,63)=21.27	**< 0.0001** F(1,154)=19.45	**< 0.0001** F(1,62)=43.56
Post-hoc test	p-value							
Spont. non-whisking vs. whisking	**0.0461** T(162)=2.294	0.2586 T(142)=1.526	0.1418 T(170)=1.817	**0.0459** T(200)=2.292	**0.0358** T(45)=2.457	0.2862 T(63)=1.483	**0.0004** T(154)=3.840	**0.0075** T(62)=3.011
WER non-whisking vs. whisking	**<0.0001** T(162)=7.488	**0.0015** T(142)=3.454	0.0673 T(170)=2.141	**<0.0001** T(200)=3.119	**0.0444** T(45)=2.369	**<0.0001** T(63)=6.676	**<0.0001** T(154)=5.097	**<0.0001** T(62)=9.513

Table S1 Spiking activity of excitatory cells when the animal was engaged in whisking or non-whisking phases

The table describes single unit activity (SUA, spikes/s) detected in cortical layers of the thalamus, POm, and VPM under the following conditions: spontaneous SUA (spont.) during whisking and non-whisking phases, as well as the whisker-evoked response (WER) under the same conditions.

Data are reported as SUA in mean ± SEM. and analysed with two-way ANOVA repeated on both factors (column factor: non-whisking vs. whisking; row factor: spontaneous/baseline vs. WER) and Bonferroni post-hoc tests. Significant effects are highlighted in bold font.

In all cortical layers, as well as in the thalamus, the spiking activity of excitatory cells increased when the animal was whisking compared to non-whisking phases (data pooled from Drd1-cre and Ntsr1-cre animals, except for L6-Drd1 and L6-Ntsr1 cells. L2/3: n = 164 spike-sorted units; L4: n = 142; L5: n = 171; L6 (not including L6-Drd1 or L6-Ntsr1 cells): n = 201; L6-Drd1: n = 46; L6-Ntsr1: n = 64); POm: n = 156; VPM: n = 63. (These data are related to Table 2 and 3, and Figure 3 of the main manuscript).

	L2/3	L4	L5	L6 non- Drd1	L6- Drd1	POm
70 ms	spikes / s	spikes / s	spikes / s	spikes / s	spikes / s	spikes / s
control	1.93 ± 0.20	1.59 ± 0.19	2.38 ± 0.29	2.18 ± 0.20	4.73 ± 0.87	7.13 ± 0.55
opto 50 mA	1.68 ± 0.24	1.23 ± 0.18	1.02 ± 0.25	1.61 ± 0.25	2.52 ± 0.97	2.52 ± 0.29
opto 100 mA	1.36 ± 0.18	1.26 ± 0.18	1.51 ± 0.24	1.27 ± 0.16	2.49 ± 0.95	2.62 ± 0.37
opto 150 mA	1.80 ± 0.22	2.09 ± 0.38	2.22 ± 0.42	2.25 ± 0.42	1.78 ± 0.78	2.91 ± 0.37
opto 200 mA	1.39 ± 0.17	2.08 ± 0.37	1.90 ± 0.32	2.57 ± 0.43	1.57 ± 0.52	3.29 ± 0.36
opto 250 mA	1.62 ± 0.21	1.85 ± 0.30	2.08 ± 0.31	2.99 ± 0.42	1.75 ± 0.31	3.79 ± 0.43
opto 300 mA	1.59 ± 0.31	1.52 ± 0.19	2.39 ± 0.37	1.69 ± 0.24	2.20 ± 0.53	3.68 ± 0.36
One-way ANOVA	p value					
	0.4477<br>F (4.150, 257.3) = 0.9331	**0.0498**<br>F (3.046, 179.7) = 2.644	0.2500<br>F (4.071, 268.7) = 1.353	**0.0056**<br>F (2.318, 180.8) = 4.942	**0.0002**<br>F (2.785, 125.3) = 7.390	**<0.0001**<br>F (4.113, 398.9) = 23.16
P value of Bonferroni post-hoc test						
ctrl vs. opto 50mA	> 0.9999<br>t(62)=1.287	0.4180<br>t(59)=1.848	0.3391<br>t(66)=1.941	>0.9999<br>t(75)=1.357	**0.0122**<br>t(45)=3.276	**<0.0001**<br>t(97)=8.880
ctrl vs. opto 100mA	> 0.9999<br>t(62)=0.06932	0.4838<br>t(59)=1.778	**0.0007**<br>t(66)=4.112	**0.0202**<br>t(75)=3.024	**0.0190**<br>t(45)=3.119	**<0.0001**<br>t(97)=8.444
ctrl vs. opto 150mA	0.0944<br>t(62)=2.483	>0.9999<br>t(59)=1.231	>0.9999<br>t(66)=0.2857	>0.9999<br>t(75)=0.6583	**0.0001**<br>t(45)=4.814	**<0.0001**<br>t(97)=7.147
ctrl vs. opto 200mA	> 0.9999<br>t(62)=0.2680	>0.9999<br>t(59)=1.234	>0.9999<br>t(66)=1.272	0.9964<br>t(75)=1.398	<0.0001<br>t(45)=6.100	**<0.0001**<br>t(97)=7.171
ctrl vs. opto 250mA	> 0.9999<br>t(62)=1.331	>0.9999<br>t(59)=0.7376	>0.9999<br>t(66)=0.8441	0.1219<br>t(75)=2.369	**<0.0001**<br>t(45)=5.071	**<0.0001**<br>t(97)=10.15
ctrl vs. opto 300mA	> 0.9999<br>t(62)=0804	>0.9999<br>t(59)=0.5962	>0.9999<br>t(66)=0.1689	>0.9999<br>t(75)=0.9200	0.0007<br>t(45)=4.222	<0.0001<br>t(97)=6.250
120 ms	spikes / s	spikes / s	spikes / s	spikes / s	spikes / s	spikes / s
ctrl	1.20 ± 0.16	2.10 ± 0.36	2.95 ± 0.45	2.47 ± 0.38	5.37 ± 1.15	6.04 ± 0.54
opto 50 mA	0.84 ± 0.13	1.27 ± 0.17	1.64 ± 0.24	1.39 ± 0.24	3.26 ± 0.97	3.00 ± 0.32
opto 100 mA	1.17 ± 0.17	0.98 ± 0.12	1.35 ± 0.21	1.59 ± 0.25	3.80 ± 0.79	3.12 ± 0.34
opto 150 mA	1.00 ± 0.13	1.04 ± 0.14	1.81 ± 0.43	1.35 ± 0.22	3.06 ± 0.74	4.09 ± 0.47
opto 200 mA	1.23 ± 0.17	1.00 ± 0.13	1.63 ± 0.29	1.75 ± 0.24	2.18 ± 0.60	3.25 ± 0.44
opto 250 mA	1.16 ± 0.15	1.27 ± 0.18	1.89 ± 0.26	1.29 ± 0.22	3.64 ± 0.71	3.35 ± 0.40
opto 300 mA	1.49 ± 0.20	2.43 ± 0.32	2.33 ± 0.33	1.99 ± 0.25	3.07 ± 0.78	5.35 ± 0.55
One-way ANOVA	p value					
	**0.0444**<br>F (3.941, 244.3) = 2.494	**<0.0001**<br>F (2.929, 172.8) = 9.658	**0.0036**<br>F (3.181, 210.0) = 2.317	**0.0061**<br>F (4.375, 341.2) = 3.518	**0.0053**<br>F (2.785, 125.3) = 7.390	**<0.0001**<br>F (3.591, 348.3) = 9.032
P value of Bonferroni post-hoc test						
ctrl vs. opto 50mA	0.0210<br>t(62)=0.8306	0.4286<br>t(59)=1.836	0.0693<br>t(66)=2.540	**0.0186**<br>t(75)=3.053	**0.0078**<br>t(45)=3.276	**0.0001**<br>t(97)=443
ctrl vs. opto 100mA	>0.9999<br>t(62)=0.4340	**0.0013**<br>t(59)=3.935	**0.0364**<br>t(66)=2.762	0.5092<br>t(75)=1.745	0.3027<br>t(45)=3.119	**0.0008**<br>t(97)=3.990
ctrl vs. opto 150mA	0.0867<br>t(62)=0.5323	**0.0079**<br>t(59)=3.372	**0.0243**<br>t(66)=2.894	**0.0012**<br>t(75)=3.905	**0.0028**<br>t(45)=4.814	**0.0266**<br>t(97)=2.913
ctrl vs. opto 200mA	>0.9999<br>t(62)=1.178	**0.0013**<br>t(59)=3.937	0.0549<br>t(66)=2.622	>0.9999<br>t(75)=1.155	**0.0010**<br>t(45)=6.100	**0.0005**<br>t(97)=4.101
ctrl vs. opto 250mA	>0.9999<br>t(62)=0.2124	0.5263<br>t(59)=1.736	**0.0467**<br>t(66)=2.677	**0.0015**<br>t(75)=3.838	**0.0012**<br>t(45)=5.071	**<0.0001**<br>t(97)=6.716
ctrl vs. opto 300mA	>0.9999<br>t(62)=1.870	**0.0449**<br>t(59)=2.770	0.7022<br>t(66)=1.571	>0.9999<br>t(75)= 0.1492	**0.0010**<br>t(45)=4.222	>0.9999<br>t(97)=0.7964

Table S2 Effect of photoinactivation of S1L6 Drd1-expressing cells on spontaneous activity
Optogenetic inactivation of L6-Drd1 cells was conducted using a 70 ms and a 120 ms long green light pulse (550 nm) at different light intensities (reported in the table in mA). The table shows spiking activity (spikes/s) for each cortical layer, L6-Drd1 and POm cells for different light intensities (L2/3 n = 63, L4 n = 60, L5 n = 67, L6 non-Drd1 n = 76, L6-Drd1 n = 46, POm n = 98 (10 animals)). Three L6 non-Drd1 cells were identified as outliers (analysed with ROUT; Q = 1%) and excluded from the analysis. In these three cells there was a large increase in spontaneous activity after L6-Drd1 inactivation.
The table describes responses in the presence (opto) or absence (control) of light stimulation. One-way ANOVA of repeated measurements and Bonferroni post-hoc tests were conducted separately on data from each cortical layer, L6-Drd1, and POm. In the main manuscript the effects of light stimulation at 250 mA are reported. Data describe SUA (single unit activity) and are reported as mean ± SEM. Significant effects are highlighted in bold font. (The data are related to Figure 1 of the main manuscript).

	L2/3	L4	L5	L6 non- Drd1
70 ms	spikes / s	spikes / s	spikes / s	spikes / s
control	1.98 ± 0.62	2.58 ± 0.66	2.45 ± 0.69	5.29 ± 2.18
opto 50 mA	1.50 ± 0.51	3.29 ± 0.91	2.37 ± 0.74	7.25 ± 2.37
opto 100 mA	1.75 ± 0.62	1.72 ± 0.25	1.63 ± 0.42	1.40 ± 0.35
opto 150 mA	1.87 ± 0.60	1.51 ± 0.34	2.49 ± 0.61	8.24 ± 4.44
opto 200 mA	1.32 ± 0.34	3.04 ± 0.83	2.42 ± 0.86	6.06 ± 3.35
opto 250 mA	1.53 ± 0.23	2.35 ± 0.73	4.25 ± 1.06	7.45 ± 4.41
opto 300 mA	2.14 ± 0.89	2.09 ± 0.60	1.86 ± 0.58	6.10 ± 2.93
One-way ANOVA	p value			
	0.7845<br>F (3.039, 21.28) = 0.3606	0.2192<br>F (3.161, 37.93) = 1.537	0.1641<br>F (3.415, 37.57) = 1.767	0.3552<br>F (1.864, 9.318) = 1.144
P value of Bonferroni post-hoc test				
ctrl vs. opto 50mA	>0.9999<br>t(7)=0.4653	>0.9999<br>t(12)=0.9023	>0.9999<br>t(11)=0.02536	>0.9999<br>t(5)=0.3623
ctrl vs. opto 100mA	>0.9999<br>t(7)=0.3297	0.7963<br>t(12)=1.613	>0.9999<br>t(11)=1.062	0.7052<br>t(5)=1.889
ctrl vs. opto 150mA	>0.9999<br>t(7)=0.1097	0.3997<br>t(12)=2.017	>0.9999<br>t(11)=0.1067	>0.9999<br>t(5)=0.06738
ctrl vs. opto 200mA	>0.9999<br>t(7)=0.7198	>0.9999<br>t(12)=0.5637	>0.9999<br>t(11)=0.01952	>0.9999<br>t(5)=0.5353
ctrl vs. opto 250mA	>0.9999<br>t(7)=0.5426	>0.9999<br>t(12)=0.4207	0.4142<br>t(11)=2.015	>0.9999<br>t(5)=0.6662
ctrl vs. opto 300mA	>0.9999<br>t(7)=0.3889	>0.9999<br>t(12)=1.235	>0.9999<br>t(11)=0.7330	>0.9999<br>t(5)=1.223
120 ms	spikes / s	spikes / s	spikes / s	spikes / s
control	2.34 ± 0.88	1.75 ± 0.39	2.84 ± 0.81	4.41 ± 2.60
opto 50 mA	2.24 ± 0.64	4.19 ± 1.61	1.17 ± 0.15	6.46 ± 3.48
opto 100 mA	1.74 ± 0.62	1.39 ± 0.27	2.14 ± 0.49	8.17 ± 3.93
opto 150 mA	2.91 ± 0.79	1.71 ± 0.25	1.82 ± 0.28	4.39 ± 2.84
opto 200 mA	2.21 ± 0.60	1.49 ± 0.33	1.76 ± 0.43	3.65 ± 2.28
opto 250 mA	2.32 ± 0.88	2.03 ± 0.35	2.15 ± 0.54	1.94 ± 0.74
opto 300 mA	1.64 ± 0.56	2.73 ± 0.66	2.52 ± 0.50	5.79 ± 1.42
One-way ANOVA	p value			
	0.4229<br>F (2.450, 17.15) = 0.9497	0.1401<br>F (1.651, 19.81) = 2.234	0.0976<br>F (3.198, 35.17) = 2.236	0.2680<br>F (1.954, 9.771) = 1.509
	P value of Bonferroni post-hoc test			
ctrl vs. opto 50mA	0.4409<br>t(7)=2.104	0.5608<br>t(12)=1.822	0.1696<br>t(11)=2.524	>0.9999<br>t(5)=1.380
ctrl vs. opto 100mA	>0.9999<br>t(7)=0.8690	>0.9999<br>t(12)=0.2273	0.9321<br>t(11)=1.526	0.8504<br>t(5)=1.743
ctrl vs. opto 150mA	0.2088<br>t(7)=2.612	>0.9999<br>t(12)=1.231	0.6347<br>t(11)=1.762	>0.9999<br>t(5)=0.9730
ctrl vs. opto 200mA	>0.9999<br>t(7)=1.095	>0.9999<br>t(12)=0.4193	0.5742<br>t(11)=1.822	>0.9999<br>t(5)=0.9564
ctrl vs. opto 250mA	>0.9999<br>t(7)=1.216	0.6011<br>t(12)=1.781	>0.9999<br>t(11)=1.197	>0.9999<br>t(5)=1.058
ctrl vs. opto 300mA	>0.9999<br>t(7)=0.3803	0.2690<br>t(12)=2.240	>0.9999<br>t(11)=0.9449	0.1249<br>t(5)=3.328

Table S3 Effect of photoinactivation of L6-Drd1 cells on spontaneous activity in interneurons
Photoinactivation of S1L6 Drd1-expressing cells was conducted using a green light pulse (550 nm) of 70 ms, or 120 ms durations, at different light intensities (reported in the table in mA). The table shows the spiking activity (spikes/s) for interneurons in each cortical layer during the application of different light intensities (L2/3 n = 8, L4 n = 13, L5 n = 12, L6 non-Drd1 n = 6; from 10 animals).
Data describe single unit activity (SUA) and are reported as mean ± SEM. One-way ANOVA of repeated measurements and Bonferroni post-hoc tests were conducted separately on data from each cortical layer. No statistically significant effects were detected. (Related to Figure 1 of main manuscript).

	L2/3	L4	L5	L6 non-Ntsr1	L6-Ntsr1	VPM	POm
70 ms	spikes / s	spikes / s	spikes / s	spikes / s	spikes / s	spikes / s	spikes / s
Ctrl 50 mA	1.55 ± 0.14	2.85 ± 0.35	3.93 ± 0.45	2.38 ± 0.24	3.13 ± 0.34	5.20 ± 0.78	5.65 ± 0.76
opto 50 mA	1.87 ± 0.22	3.39 ± 0.67	4.34 ± 0.45	2.18 ± 0.21	1.41 ± 0.17	3.17 ± 0.51	3.07 ± 0.50
opto 100 mA	1.44 ± 0.15	2.65 ± 0.50	3.06 ± 0.38	2.03 ± 0.24	1.48 ± 0.22	3.33 ± 0.59	3.68 ± 0.69
opto 150 mA	1.82 ± 0.22	3.09 ± 0.46	4.13 ± 0.47	2.48 ± 0.22	1.33 ± 0.21	3.44 ± 0.58	3.67 ± 0.79
opto 200 mA	1.33 ± 0.13	2.11 ± 0.29	3.59 ± 0.42	1.95 ± 0.18	1.22 ± 0.19	3.42 ± 0.61	2.66 ± 0.39
opto 250 mA	1.32 ± 0.14	2.17 ± 0.35	2.94 ± 0.39	1.87 ± 0.14	1.01 ± 0.14	3.30 ± 0.59	3.95 ± 0.57
opto 300 mA	1.76 ± 0.26	3.59 ± 0.62	5.12 ± 0.53	3.31 ± 0.49	1.67 ± 0.26	3.10 ± 0.65	3.41 ± 0.47
One-way ANOVA	p value (Bonferroni corrected)						
	0.0846<br>F (4.087, 408.7) = 2.054	0.0888<br>F (3.735, 302.6) = 2.074	**0.0004**<br>F (3.916, 403.4) = 5.311	0.0099<br>F (2.467, 288.7) = 4.234	**<0.0001**<br>F (3.876, 244.2) = 6.319	**0.0025**<br>F (3.948, 244.8) =4.264	**<0.0001**<br>F (4.080, 232.6) =8.099
P value of Bonferroni post-hoc test							
ctrl vs. opto 50mA	>0.9128<br>t(100)=1.443	>0.9999<br>t(82)=0.8225	>0.9999<br>t(103)=0.4466	>0.9999<br>t(118)=0.4781	**0.0010**<br>t(63)=4.005	**0.0091**<br>t(62)=3.319	**<0.0001**<br>t(57)=5.259
ctrl vs. opto 100mA	>0.9999<br>t(100)=0.3873	>0.9999<br>t(82)=0.3375	0.0628<br>t(103)=2.608	>0.9999<br>t(118)=1.042	**0.0142**<br>t(63)=3.168	**0.0099**<br>t(62)=3.293	**0.0005**<br>t(57)=4.255
ctrl vs. opto 150mA	0.9999<br>t(100)=1.294	>0.9999<br>t(82)=0.5004	>0.9999<br>t(103)=0.04272	>0.9999<br>(118)=0.7082	**0.0025**<br>t(63)=3.723	**0.0138**<br>t(62)=3.179	**0.0084**<br>t(57)=3.359
ctrl vs. opto 200mA	>0.9999<br>t(100)=1.117	0.3625<br>t(82)=1.904	>0.9999<br>t(103)=1.162	0.8692<br>t(118)=1.468	**0.0008**<br>t(63)=4.082	**0.0254**<br>t(62)=2.970	**<0.0001**<br>t(57)=5.110
ctrl vs. opto 250mA	>0.9999<br>t(100)=0.9864	>0.9999<br>t(82)=1.373	**0.0029**<br>t(103)=3.607	0.3777<br>t(118)=1.877	**<0.0001**<br>t(63)=6.574	**<0.0001**<br>t(62)=6.155	**0.0001**<br>t(57)=6.764
ctrl vs. opto 300mA	>0.9999<br>t(100)=0.9489	>0.9999<br>t(82)=1.134	0.4083<br>t(103)=1.844	0.3376<br>t(118)=1.928	0.1312<br>t(63)=2.351	**0.0097**<br>t(62)=3.299	**0.0001**<br>t(57)=4.673
120 ms	spikes / s	spikes / s	spikes / s	spikes / s	spikes / s	spikes / s	spikes / s
Ctrl 50 mA	1.25 ± 0.14	2.90 ± 0.42	3.81 ± 0.48	2.20 ± 0.27	6.22 ± 0.95	6.74 ± 1.21	6.46 ± 0.69
opto 50 mA	1.47 ± 0.19	2.61 ± 0.43	3.85 ± 0.60	2.41 ± 0.24	1.35 ± 0.26	5.69 ± 1.38	3.69 ± 0.55
opto 100 mA	1.54 ± 0.21	2.02 ± 0.29	3.35 ± 0.49	2.06 ± 0.23	1.29 ± 0.27	5.46 ± 1.20	3.83 ± 0.56
opto 150 mA	1.58 ± 0.19	2.52 ± 0.38	3.80 ± 0.57	2.06 ± 0.20	1.55 ± 0.31	4.18 ± 1.06	3.06 ± 0.46
opto 200 mA	1.46 ± 0.18	2.23 ± 0.35	3.21 ± 0.50	1.87 ± 0.19	1.35 ± 0.31	4.51 ± 1.19	2.38 ± 0.41
opto 250 mA	1.77 ± 0.21	2.85 ± 0.43	3.85 ± 0.53	2.28 ± 0.21	1.15 ± 0.24	4.35 ± 0.69	3.50 ± 0.52
opto 300 mA	1.33 ± 0.18	3.34 ± 0.55	3.97 ± 0.35	2.64 ± 0.24	2.63 ± 0.40	3.95 ± 0.64	3.41 ± 0.46
One-way ANOVA	p value (Bonferroni corrected)						
	0.2863<br>F (3.706, 370.6) = 1.260	0.0982<br>F (3.591, 290.9) = 2.027	0.2333<br>F (3.554, 366.0) = 1.414	**0.0062**<br>F (4.438, 519.3) = 3.466	**<0.0001**<br>F (1.393, 87.73) = 22.80	0.1824<br>F (2.431, 150.7) = 1.684	**<0.0001**<br>F (3.914, 223.1) = 12.03
P value of Bonferroni post-hoc test							
ctrl vs. opto 50mA	>0.9999<br>t(100)=0.5064	>0.9999<br>t(82)=0.4234	0.4486<br>t(103)=1.800	0.0810<br>t(118)=2.508	**<0.0001**<br>t(63)=5.064	>0.9999<br>t(62)=0.4031	**0.0001**<br>t(57)=4.602
ctrl vs. opto 100mA	>0.9999<br>t(100)=0.7295	>0.9999<br>t(82)=1.146	>0.9999<br>t(103)=0.7347	>0.9999<br>t(118)=0.8345	**<0.0001**<br>t(63)=5.155	>0.9999<br>t(62)=0.6708	**0.0023**<br>t(57)=3.775
ctrl vs. opto 150mA	>0.9999<br>t(100)=0.9851	>0.9999<br>t(82)=0.2810	0.3527<br>t(103)=1.911	>0.9999<br>t(118)=0.9264	**<0.0001**<br>t(63)=4.954	0.3281<br>t(62)=1.958	**<0.0001**<br>t(57)=5.252
ctrl vs. opto 200mA	>0.9999<br>t(100)=0.4851	>0.9999<br>t(82)=0.6286	>0.9999<br>t(103)=0.3123	>0.9999<br>t(118)=0.02153	**<0.0001**<br>t(63)=5.236	0.6943<br>t(62)=1.595	**<0.0001**<br>t(57)=6.533
ctrl vs. opto 250mA	0.3436<br>t(100)=1.923	>0.9999<br>t(82)=1.246	0.0524<br>t(103)=2.673	0.1440<br>t(118)=2.287	**<0.0001**<br>t(63)=5.331	**<0.0001**<br>t(62)=5.267	**<0.0001**<br>t(57)=7.199
ctrl vs. opto 300mA	>0.9999<br>t(100)=0.06094	0.7778<br>t(82)=1.531	0.5499<br>t(103)=1.703	**0.0405**<br>t(118)=2.758	**0.0003**<br>t(63)=4.383	**0.0156**<br>t(62)=3.139	**0.0002**<br>t(57)=4.500

Table S4 Effect of photoinactivation of S1L6 Ntsr1-expressing cells on spontaneous activity
Optogenetic inactivation of Ntsr1-expressing cells in S1L6 was conducted using a green light pulse (550 nm) of 70 ms, or 120 ms durations, at different light intensities (reported in the table in mA). The table shows spiking activity (spikes/s) for each cortical layer, L6-Ntsr1, VPM and POm cells for different light intensities (L2/3 n = 101, L4 n = 83, L5 n = 104, L6 non-Ntsr1 n = 119, L6-Ntsr1 n = 64, VPM n = 63, POm n = 58 (12 animals)). Data describe single-unit activity (SUA) and are reported as mean ± SEM. One-way ANOVA of repeated measurements and Bonferroni post-hoc tests were conducted separately on data from each cortical layer, L6-Ntsr1, VPM and POm. In the main text the results are exemplified with the data for 250 mA. Significant effects are highlighted in bold font. (Related to Table 3, Figure 2 of main manuscript).

	L2/3	L4	L5	L6 non-Ntsr1
70 ms	spikes / s	spikes / s	spikes / s	spikes / s
control	1.87 ± 0.72	2.14 ± 0.56	7.23 ± 3.24	2.30 ± 0.61
opto 50 mA	1.93 ± 0.65	2.58 ± 1.09	6.37 ± 3.09	2.12 ± 0.34
opto 100 mA	0.94 ± 0.26	2.45 ± 1.12	7.35 ± 3.09	1.71 ± 0.36
opto 150 mA	4.22 ± 1.57	2.39 ± 0.61	6.35 ± 3.37	3.91 ± 1.13
opto 200 mA	1.01 ± 0.33	2.07 ± 0.78	5.85 ± 2.69	2.48 ± 0.66
opto 250 mA	1.30 ± 0.44	1.27 ± 0.29	4.36 ± 1.50	2.40 ± 0.70
opto 300 mA	1.57 ± 1.08	3.20 ± 0.50	6.33 ± 2.10	4.22 ± 1.27
One-way ANOVA	p value			
	0.2035<br>F (2.014, 16.11) = 1.760	0.3810<br>F (1.862, 24.21) = 0.9895	0.6457<br>F (2.840, 28.40) = 0.5458	0.1894<br>F (2.534, 27.87) = 1.731
p value of Bonferroni post-hoc test				
ctrl vs. opto 50mA	>0.9999<br>t(8)=0.6474	>0.9999<br>t(13)=0.06969	>0.9999<br>t(10)=0.03724	>0.9999<br>t(11)=0.5513
ctrl vs. opto 100mA	>0.9999<br>t(8)=1.473	>0.9999<br>t(13)=0.2057	>0.9999<br>t(10)=0.4408	0.7643<br>t(11)=1.649
ctrl vs. opto 150mA	0.9530<br>t(8)=1.554	>0.9999<br>t(13)=0.3582	>0.9999<br>t(10)=0.04384	>0.9999<br>t(11)=1.078
ctrl vs. opto 200mA	>0.9999<br>t(8)=0.8723	>0.9999<br>t(13)=0.8515	>0.9999<br>t(10)=0.4498	>0.9999<br>t(11)=0.05790
ctrl vs. opto 250mA	>0.9999<br>t(8)=0.4328	0.1373<br>t(13)=2.580	0.8978<br>t(10)=1.561	>0.9999<br>t(11)=0.08528
ctrl vs. opto 300mA	>0.9999<br>t(8)=0.009751	>0.9999<br>t(13)=1.260	>0.9999<br>t(10)=0.06654	>0.9999<br>t(11)=1.304
120 ms	spikes / s	spikes / s	spikes / s	spikes / s
control	1.20 ± 0.26	2.04 ± 0.62	5.04 ± 2.57	2.95 ± 1.09
opto 50 mA	1.45 ± 0.27	1.97 ± 0.49	7.44 ± 3.28	3.14 ± 1.27
opto 100 mA	1.28 ± 0.15	1.12 ± 0.28	5.54 ± 2.23	2.58 ± 0.92
opto 150 mA	1.25 ± 0.21	1.35 ± 0.22	6.83 ± 1.93	2.91 ± 1.16
opto 200 mA	0.97 ± 0.27	1.43 ± 0.31	4.73 ± 1.79	2.06 ± 0.38
opto 250 mA	2.76 ± 1.05	2.91 ± 0.85	5.75 ± 1.83	2.99 ± 0.77
opto 300 mA	1.10 ± 0.28	3.74 ± 0.73	5.51 ± 1.96	3.19 ± 0.75
One-way ANOVA	p value			
	0.2657<br>F (1, 8) = 1.432	0.0532<br>F (1.704, 22.16) = 3.529	0.1123<br>F (2.155, 21.55) = 2.390	0.7640<br>F (2.503, 27.53) = 0.3358
	p value of Bonferroni post-hoc test			
ctrl vs. opto 50mA	>0.9999<br>t(8)=0.6503	>0.9999<br>t(13)=0.8030	0.5035<br>t(10)=1.808	>0.9999<br>t(11)=0.6731
ctrl vs. opto 100mA	>0.9999<br>t(8)=0.02919	0.7700<br>t(13)=1.624	0.4054<br>t(10)=1.940	>0.9999<br>t(11)=0.3830
ctrl vs. opto 150mA	>0.9999<br>t(8)=0.2528	>0.9999<br>t(13)=1.406	0.0833<br>t(10)=2.870	>0.9999<br>t(11)=0.5372
ctrl vs. opto 200mA	>0.9999<br>t(8)=0.6810	>0.9999<br>t(13)=1.334	0.9825<br>t(10)=1.384	>0.9999<br>t(11)=0.1079
ctrl vs. opto 250mA	0.0699<br>t(8)=2.644	>0.9999<br>t(13)=0.01421	0.3062<br>t(10)=2.108	0.6263<br>t(11)=1.770
ctrl vs. opto 300mA	>0.9999<br>t(8)=0.5868	>0.9999<br>t(13)=1.118	0.9999<br>t(10)=0.189	0.1779<br>t(11)=2.497

Table S5 Effect of photoinactivation of S1L6 Ntsr1-expressing cells on spontaneous activity of interneurons

Optogenetic inactivation of Ntsr1 cells was conducted using a 70 ms and a 120 ms long green light pulse (550 nm) at different light intensities (reported in the table in mA). The table shows the spiking activity (spikes/s) for interneurons in each cortical layer for different light intensities (L2/3 n = 9, L4 n = 14, L5 n = 11, L6 non-Ntsr1 n = 12; from 12 animals). Data describe single-unit activity (SUA) and are reported as mean ± SEM.

One-way ANOVA of repeated measurements and Bonferroni post-hoc tests done separately on data from each cortical layer. No statistically significant effects were detected. (Related to Figure 2 of main manuscript).

	L2/3	L4	L5	L6 non- Drd1
	spikes/s	spikes/s	spikes/s	spikes/s
	No whisking	No whisking	No whisking	No whisking
spont. control	8.61 ± 1.24	5.39 ± 2.44	2.70 ± 0.82	11.38 ± 3.86
spont. opto 50ms	7.09 ± 1.58	4.28 ± 1.50	2.56 ± 1.05	10.93 ± 4.36
spont. opto 100ms	4.68 ± 1.40	4.71 ± 0.93	1.93 ± 0.80	11.07 ± 4.30
WER control	49.47 ± 9.36	48.31 ± 8.66	50.22 ± 12.89	40.21 ± 12.19
WER + 50ms opto	46.23 ± 9.33	50.17 ± 9.55	46.55 ± 13.06	39.71 ± 12.65
WER + 100ms opto	43.18 ± 11.74	51.40 ± 9.36	47.03 ± 11.78	39.72 ± 10.97
Two-way ANOVA	p value	p value	p value	p value
Spont-resp	**0.0046** F(1,7)=16.79	**0.0002** F(1,12)=26.40	**0.0031** F(1,11)=14.23	**0.0357** F(1,5)=8.143
Ctrl.-50ms-100ms	0.2643 F(2,14)=1.47	0.8658 F(2,24)=0.1450	0.5375 F(2,22)=0.6386	0.9832 F(2,10)=0.01692
Interaction	0.9347 F(2,14)=0.06781	0.7905 F(2,24)=0.2374	0.6410 F(2,22)=0.4539	0.9987 F(2,10)=0.001302
Bonferroni Post-hoc test	p value	p value	p value	p value
spont.: control vs. 50ms opto	>0.9999	>0.9999	>0.9999	>0.9999
WER: ctrl vs. 50ms opto	0.8315	>0.9999	0.3693	>0.9999
spont.: ctrl vs. 100ms opto	>0.9999	>0.9999	>0.9999	>0.9999
WER: ctrl vs. 100ms	0.4018	0.9123	0.4917	>0.9999
	spikes/s	spikes/s	spikes/s	spikes/s
	whisking	whisking	whisking	whisking
baseline control	7.94 ± 3.46	5.38 ± 1.40	4.88 ± 0.79	8.54 ± 4.18
basel. opto 50ms	8.88 ± 3.07	3.54 ± 1.53	5.93 ± 1.36	11.22 ± 5.24
basel. opto 100ms	2.26 ± 1.40	3.88 ± 1.09	3.48 ± 0.85	4.22 ± 1.66
WER control	43.47 ± 11.54	53.69 ± 10.88	43.76 ± 11.42	37.51 ± 9.16
WER + 50ms opto	40.51 ± 9.08	57.88 ± 10.67	42.21 ± 13.65	40.30 ± 13.28
WER + 100ms opto	43.42 ± 11.60	49.29 ± 9.01	46.03 ± 14.40	39.52 ± 9.92
Two-way ANOVA	p value	p value	p value	p value
Spont-resp	**0.0123** F(1,7)=11.20	**0.0003** F(1,12)=25.69	**0.0107** F(1,11)=9.427	**0.0174** F(1,5)=12.19
Ctrl.-50ms-100ms	0.6990 F(2,14)=0.3674	0.3477 F(2,24)=1.104	0.9591 F(2,22)=0.04180	0.5612 F(2,10)=0.6124
Interaction	0.1968 F(2,14)=1.830	0.3609 F(2,24)=1.064	0.5572 F(2,22)=0.6006	0.3654 F(2,10)=1.115
Bonferroni Post-hoc test	p value	p value	p value	p value
basel.: control vs. 50ms opto	>0.9999	>0.9999	>0.9999	0.9645
WER: ctrl vs. 50ms opto	0.2617	>0.9999	>0.9999	0.9272
basel.: ctrl vs. 100ms opto	0.8349	>0.9999	>0.9999	0.5314
WER: ctrl vs. 100ms	>0.9999	0.9123	>0.9999	>0.9999

Table S6 Registration in interneurons of cell activity during monitoring of the effect of photoinactivation of L6-Drd1 cells on whisker-evoked activity during non-whisking and whisking phases

The table shows spiking activity (spikes/s) for interneurons in each cortical layer for spontaneous (spont.), baseline (basel.) activity and whisker-evoked responses (WER), in the presence (opto, light intensity 250 mA) or absence (ctrl.) of photoinactivation of L6-Drd1 cells: (L2/3 n = 8, L4 n = 13, L5 n = 12, L6 non-Drd1 n = 6; form 10 animals). Data describe single-unit activity (SUA) and are reported as mean ± SEM. A two-way ANOVA repeated on both factors with a Bonferroni post-hoc test was conducted separately on data from each cortical layer. Significant effects are highlighted in bold font.

	L2/3	L4	L5	L6 non-Ntsr1
	spikes/s	spikes/s	spikes/s	spikes/s
	No whisking	No whisking	No whisking	No whisking
spont. control	5.10 ± 2.68	4.04 ± 1.15	6.14 ± 3.43	3.66 ± 1.67
spont. opto 50ms	4.43 ± 2.64	4.67 ± 1.69	3.64 ± 1.52	3.84 ± 1.43
spont. opto 100ms	5.14 ± 2.67	3.56 ± 1.02	4.05 ± 2.50	4.26 ± 1.83
WER control	31.16 ± 7.26	36.90 ± 6.93	59.60 ± 14.87	31.17 ± 8.87
WER + 50ms opto	35.94 ± 11.17	34.62 ± 6.65	59.31 ± 16.30	30.58 ± 8.60
WER + 100ms opto	30.87 ± 7.41	40.54 ± 6.58	55.40 ± 13.79	34.33 ± 8.84
Two-way ANOVA	p value	p value	p value	p value
Spont-resp	**0.0125** F(1,8)=10.29	**0.0002** F(1,13)=27.26	**0.0021** F(1,10)=16.88	**0.0058** F(1,11)=11.66
Ctrl.-50ms-100ms	0.6595 F(2,16)=0.4273	0.5247 F(2,26)=0.5247	0.5503 F(2,20)=0.6155	0.5885 F(2,22)=0.5432
Interaction	0.5789 F(2,16)=0.5657	0.2536 F(2,26)=1.447	0.7463 F(2,20)=0.2969	0.6107 F(2,22)=0.5043
Bonferroni Post-hoc test	p value	p value	p value	p value
spont.: control vs. 50ms opto	>0.9999	>0.9999	>0.9999	>0.9999
WER: ctrl vs. 50ms opto	0.5709	0.8872	>0.9999	>0.9999
spont.: ctrl vs. 100ms opto	>0.9999	>0.9999	>0.9999	>0.9999
WER: ctrl vs. 100ms	>0.9999	0.4529	0.6065	0.4239
	spikes/s whisking	spikes/s whisking	spikes/s whisking	spikes/s whisking
baseline control	7.24 ± 2.44	4.95 ± 1.31	8.91 ± 2.43	4.43 ± 1.54
basel. opto 50ms	5.74 ± 1.46	3.34 ± 0.74	7.00 ± 1.75	6.32 ± 1.62
basel. opto 100ms	2.86 ± 1.04	3.24 ± 0.55	5.23 ± 1.53	5.46 ± 1.40
WER control	31.29 ± 5.46	43.90 ± 8.03	80.35 ± 16.95	35.16 ± 8.89
WER + 50ms opto	30.15 ± 5.07	39.92 ± 6.54	74.33 ± 18.01	45.27 ± 9.76
WER + 100ms opto	28.09 ± 5.62	43.11 ± 7.46	74.00 ± 17.23	46.33 ± 10.10
Two-way ANOVA	p value	p value	p value	p value
Spont-resp	**0.0004** F(1,8)=33.20	**<0.0001** F(1,13)=33.77	**0.0016** F(1,10)=18.35	**0.0012** F(1,11)=18.87
Ctrl.-50ms-100ms	0.2698 F(2,16)=1.423	0.6920 F(2,26)=0.3734	0.0500 F(2,20)=3.493	0.1103 F(2,22)=2.441
Interaction	0.9552 F(2,16)=0.04602	0.8577 F(2,26)=0.1544	0.5119 F(2,20)=0.6926	0.2693 F(2,22)=1.394
Bonferroni Post-hoc test	p value	p value	p value	p value
basel.: control vs. 50ms opto	>0.9999	>0.9999	0.9107	>0.9999
WER: ctrl vs. 50ms opto	0.2787	0.7300	0.0524	0.0749
basel.: ctrl vs. 100ms opto	>0.9999	>0.9999	0.3160	>0.9999
WER: ctrl vs. 100ms	0.5444	>0.9999	0.0395	0.0456

Table S7 Effect of photoinactivation of S1L6 Ntsr1-expressing cells on interneurons during whisker-evoked activity in non- whisking and whisking phases

The table shows spiking activity (spikes/s) for interneurons in each cortical layer during spontaneous (spont.), baseline (basel.) activity and whisker-evoked responses (WER) in the presence (opto, light intensity 250 mA) or absence (ctrl.) of photoinactivation of L6-Ntsr1 cells: (L2/3 n = 9, L4 n = 14, L5 n = 11, L6 non-Ntsr1 n = 12; form 12 animals). Data describe single-unit activity (SUA) and are reported as mean ± SEM.

Two-way ANOVA repeated on both factors with Bonferroni post-hoc test was conducted separately on data from each cortical layer. Significant effects are highlighted in bold font.

	L2/3	L4	L5	L6	L6-Drd1	L6-Ntsr1	POm	VPM
Refractory period (ms)	9.35 ± 0.47	6.68 ± 0.24	7.17 ± 0.19	7.90 ± 0.23	8.56 ± 0.53	7.16 ± 0.33	6.45 ± 0.20	8.19 ± 0.44

Table S8 Refractory periods of recorded neurons
The table shows the refractory periods (mean ± SEM) of recorded neurons (in ms). The refractory period in the autocorrelogram was estimated as the peak from the hazard function (Bar-Gad et al., 2001) and calculated with Neuroexplorer software (Nex Technologies, Colorado Springs, CO, USA)). L2/3 (mean ± SD; n = 181 spike sorted units), for L4 (n = 169), for L5 (n = 194), for L6 (n = 219), for L6-Drd1 (n = 46), for L6-Ntsr1 (n = 64), for POm (n = 156) and VPM (n = 63). The data are from both Drd1 and Ntsr1 animals and collected when the animal was not whisking.

**Drd1-Cre Experiment (1-10)**	**1**	**2**	**3**	**4**	**5**	**6**	**7**	**8**	**9**	**10**		
Opto non-whisking	30	27	25	22	20	27	28	20	21	30		
Opto whisking	20	20	23	27	20	22	30	20	21	20		
WER ctrl. non-whisking	30	28	20	23	30	26	20	20	26	26		
WER opto non- whisking	28	25	22	30	30	20	20	27	24	20		
WER ctrl. whisking	24	28	20	24	22	30	25	27	26	28		
WER opto whisking	22	30	26	22	24	29	30	21	20	22		
**Ntsr1-Cre Experiment (1-12)**	**1**	**2**	**3**	**4**	**5**	**6**	**7**	**8**	**9**	**10**	**11**	**12**
Opto non- whisking	22	28	27	22	25	26	20	22	27	22	22	22
Opto whisking	22	20	22	22	19	20	25	20	17	20	18	21
WER ctrl. non- whisking	24	25	30	22	30	24	22	28	29	24	30	25
WER opto non- whisking	30	24	30	30	20	25	20	20	22	23	26	25
WER ctrl. whisking	20	30	26	30	22	24	29	26	24	22	27	30
WER opto whisking	23	27	30	25	22	20	30	30	26	28	22	30

Table S9 Number of trials recorded for each cell and experiment
The table shows each of the recorded protocols in Drd1-Cre and Ntsr1-Cre animals. For Drd1-cre mice, 10 experiments were conducted. In in Ntsr1-Cre mice 12 experiments were conducted.
Ctrl.: control, Opto: photoinactivation of arachaerhodopsin in S1L6, WER: whisker-evoked response.

Protocol	L6-Drd1 70 ms	L6-Ntsr1 70 ms	L6-Drd1 120 ms	L6-Ntsr1 120 ms
	opto minus spont	opto minus spont	opto minus spont	opto minus spont
	spikes/s	spikes/s	spikes/s	spikes/s
50 mA	-1.67 ± 0.33	-1.19 ± 0.22	-2.16 ± 0.68	-4.77 ± 1.04
100 mA	-1.56 ± 0.46	-1.42 ± 0.21	-2.34 ± 0.66	-5.26 ± 1.01
150 mA	-1.50 ± 0.44	-1.27 ± 0.15	-3.99 ± 0.67	-4.98 ± 1.02
200 mA	-1.26 ± 0.34	-2.14 ± 0.43	-2.52 ± 0.63	-5.46 ± 1.02
250 mA	-2.94 ± 0.37	-1.45 ± 0.22	-3.08 ± 0.42	-5.54 ± 1.04
300 mA	-2.42 ± 0.36	-3.19 ± 0.39	-2.14 ± 0.42	-1.99 ± 0.25
	p-value		p-value	
Main factor: cell type L6-Drd1 vs. L6-Ntsr1	0.6824 F(1,108)=0.1683		0.0733 F(1,108)=3.272	

Table S10 Comparison of the effect of photoinactivation in S1L6 Drd1-expressing and S1L6 Ntsr1-expressing cells
The value in the table reports spiking activity during optogenetic photoinactivation (“opto”) minus spiking without application of light pulses (“spont”) in spikes/s from Table S1 and S3. S1L6-Drd1 corresponds to n = 46 cells and S1L6-Ntsr1 corresponds to n = 64 cells. Data describe single-unit activity (SUA) and are reported as mean ± SEM. “X mA” refers to light intensity.
Statistical analysis: Two-way ANOVA repeated on one factor (light intensity). No statistically significant effects were detected.

Data S1: Information about analysis strategy reported in Figure S5

Comparison of the absolute change in thalamic whisker-evoked activity during non-whisking and whisking episodes, elicited by photoinactivation of the two different cell populations in S1L6

To compare the absolute whisker-evoked response (WER) value in the presence, or absence, of photoinactivation when the animal was engaged in whisking or non-whisking activity, a linear regression model was used (linear regression of x: WER without photoinactivation vs. y: WER with photoinactivation; **Fig. S5**) and the slopes were then compared. The slope is < 1, because the data show that with L6- photoinactivation the whisker-evoked response is smaller. The aim was to compare how much smaller the effect was, when WER, during whisking and non- whisking episodes, was compared: The smaller the slope, the larger is the effect of photo-inactivation on the whisker-evoked response (**Fig. S5**). This analysis shows that when the animal was whisking, there was a larger effect of photoinactivation of S1L6-Drd1 cells on WER in POm, compared to when the animal was not whisking, as indicated by the smaller slope ($p = 0.0003594$; $F(1,188) = 13.2091$) of the linear regression line (non-whisking: $y = 0.7736x + 2.482$ ($R^2 = 0.7736$); whisking: $y = 0.5370x + 8.555$ ($R^2 = 0.5748$)). Interestingly, during photoinactivation of S1L6-Ntsr1 cells, the linear regression slope in POm was the same during the whisking and non-whisking episodes ($p = 0.1446$; $F(1,114) = 2.07899$; non-whisking: $y = 0.8744x + 0.56002$ ($R^2 = 0.6790$); whisking: $y = 0.7029x + 4.103$ ($R^2 = 0.5642$). Thus, S1L6-Ntsr1cell photoinactivation caused a similar absolute WER spiking decrease in POm during non-whisking and whisking phases.

Data S2: Analysis of the prolonged WER in POm

The prolonged response detected in POm did not represent a clear second peak, as was the case for VPM, and appeared between 50 ms and 150 ms after whisker stimulation onset. Moreover, this response was not detectable in all recorded cells.

Nevertheless, analysis of the prolonged response in POm cells that exhibited this effect, revealed the following: Photoinactivation of Drd1-expressing S1L6 cells had no effect on the prolonged POm response during non- whisking episodes (control(ctrl) response: 32.41 ± 2.30 spikes/s; response optogenetic (opto) response: 29.55 ± 2.14 spikes/s; two-way ANOVA repeated on both factors, $p = 0.3101$, $n = 79$).

Photoinactivation of Drd1-expressing S1L6 cells during whisking significantly reduced the prolonged POm response (response ctrl: 45.83 ± 4.77 spikes/s; response opto: 31.30 ± 3.44 spikes/s; two-way ANOVA repeated on both factors, $p < 0.0001$, $n = 79$).

POm cells recorded in Ntsr1-cre mice *only* showed this prolonged response when the animal was whisking. Here too, not all the recorded cells showed this prolonged response. Photoinactivation of Ntsr1-expressing S1L6 cells during whisking, significantly reduced the prolonged POm response (response ctrl: 43.89 ± 6.94 spikes/s; response opto: 34.37 ± 4.14 spikes/s; two-way ANOVA repeated on both factors, $p < 0.0001$, $n = 23$).

Supplemental References

1. Fanselow, E. E., Sameshima, K., Baccala, L. A., & Nicolelis, M. A. (2001). Thalamic bursting in rats during different awake behavioral states. *Proceedings of the National Academy of Sciences of the United States of America*, *98*(26), 15330–15335. https://doi.org/10.1073/pnas.261273898

2. Franklin KBJ and Paxinos G. (2013). Paxinos and Franklin's The mouse brain in stereotaxic coordinates. *Academic Press, an Imprint of Elsevier.*, *4*.

3. Perkon, I., Košir, A., Itskov, P. M., Tasič, J., & Diamond, M. E. (2011). Unsupervised quantification of whisking and head movement in freely moving rodents. *Journal of Neurophysiology*, *105*(4), 1950–1962. https://doi.org/10.1152/jn.00764.2010
